# Derivedness Index for Estimating Degree of Phenotypic Evolution of Embryos: A Study of Comparative Transcriptomic Analyses of Chordates and Echinoderms

**DOI:** 10.3389/fcell.2021.749963

**Published:** 2021-11-26

**Authors:** Jason Cheok Kuan Leong, Yongxin Li, Masahiro Uesaka, Yui Uchida, Akihito Omori, Meng Hao, Wenting Wan, Yang Dong, Yandong Ren, Si Zhang, Tao Zeng, Fayou Wang, Luonan Chen, Gary Wessel, Brian T. Livingston, Cynthia Bradham, Wen Wang, Naoki Irie

**Affiliations:** ^1^ Department of Biological Sciences, Graduate School of Science, The University of Tokyo, Tokyo, Japan; ^2^ State Key Laboratory of Genetic Resources and Evolution, Kunming Institute of Zoology, Chinese Academy of Sciences, Kunming, China; ^3^ RIKEN Center for Biosystems Dynamics Research (BDR), Kobe, Japan; ^4^ Universal Biology Institute, The University of Tokyo, Tokyo, Japan; ^5^ Sado Island Center for Ecological Sustainability, Niigata University, Niigata, Japan; ^6^ Shanghai Institute of Biochemistry and Cell Biology, Center for Excellence in Molecular Cell Science, Chinese Academy of Sciences, Shanghai, China; ^7^ Key Laboratory of Systems Biology, Hangzhou Institute for Advanced Study, University of Chinese Academy of Sciences, Chinese Academy of Sciences, Hangzhou, China; ^8^ Providence Institute of Molecular Oogenesis, Brown University, Providence, RI, United States; ^9^ Department of Biological Sciences, California State University, Long Beach, CA, United States; ^10^ Department of Biology, Boston University, Boston, MA, United States; ^11^ School of Ecology and Environment, Northwestern Polytechnical University, Xi’an, China

**Keywords:** derivedness, evo-devo, phenotypic evolution, phylotypic period, chordates, echinoderms

## Abstract

Species retaining ancestral features, such as species called living fossils, are often regarded as less derived than their sister groups, but such discussions are usually based on qualitative enumeration of conserved traits. This approach creates a major barrier, especially when quantifying the degree of phenotypic evolution or degree of derivedness, since it focuses only on commonly shared traits, and newly acquired or lost traits are often overlooked. To provide a potential solution to this problem, especially for inter-species comparison of gene expression profiles, we propose a new method named “derivedness index” to quantify the degree of derivedness. In contrast to the conservation-based approach, which deals with expressions of commonly shared genes among species being compared, the derivedness index also considers those that were potentially lost or duplicated during evolution. By applying our method, we found that the gene expression profiles of penta-radial phases in echinoderm tended to be more highly derived than those of the bilateral phase. However, our results suggest that echinoderms may not have experienced much larger modifications to their developmental systems than chordates, at least at the transcriptomic level. In vertebrates, we found that the mid-embryonic and organogenesis stages were generally less derived than the earlier or later stages, indicating that the conserved phylotypic period is also less derived. We also found genes that potentially explain less derivedness, such as Hox genes. Finally, we highlight technical concerns that may influence the measured transcriptomic derivedness, such as read depth and library preparation protocols, for further improvement of our method through future studies. We anticipate that this index will serve as a quantitative guide in the search for constrained developmental phases or processes.

## 1 Introduction

Considering the fact that species of different lineages have spent exactly the same geological time since the split from their common ancestor (Baum and Smith, 2012), it can be said that they are equally evolved. However, various factors, such as different generation turnover times (Martin and Palumbi, 1993; Li et al., 1996) and population sizes (Woolfit, 2009; Lynch et al., 2016), have led to different biological times spent by them in different lineages (Bromham and Penny, 2003; Baer et al., 2007; Lanfear et al., 2010; Gaut et al., 2011). This means that the evolutionary speed of each species and lineages differs from each other, as indicated by the different genome evolutionary rates (Green et al., 2014; Urry et al., 2016). Species called living fossils, such as coelacanth (Amemiya et al., 2013) and tuatara (Miller et al., 2009), are good examples because they retain a variety of ancestral or conserved traits and have slower evolutionary rates in their genomes than their sister groups (Amemiya et al., 2013; Gemmell et al., 2020). Similarly, phenotypic changes during evolution also differ among different traits even within the same species; some traits, such as basic anatomical features, or the body plan for each animal phylum, remain strictly conserved through hundreds of millions of years (Arthur, 1997; Erwin et al., 2011), while body size or coat colors appear to change rather frequently (Stern, 2001; Hoekstra, 2006). Recent studies have demonstrated that mid-embryonic, organogenesis stages (or phylotypic period in the developmental hourglass model (Duboule, 1994)) of animals (such as vertebrates (Hazkani‐Covo et al., 2005; Irie and Sehara-Fujisawa, 2007; Domazet-Lošo and Tautz, 2010; Irie and Kuratani, 2011; Wang et al., 2013; Hu et al., 2017), *Drosophila* species (Kalinka et al., 2010), nematodes (Levin et al., 2012), and molluscs (Xu et al., 2016)) are evolutionarily more conserved than their earlier or later developmental stages. This implies that conserved stages or traits are more ancestral; however, it has to be noted that conservation may not necessarily indicate that these traits are “less derived” than others, retaining more ancestral states. This is because “conservation” generally focuses on traits, genes, or genomic sequences commonly shared among the species being compared, and those that are lost or newly acquired during evolution are often excluded. In other words, the degree of changes accumulated during evolution or degree of “derivedness,” may not be effectively measured by estimations using only commonly shared features.

This causes a variety of ambiguities in understanding phenotypic evolution. For example, we still cannot determine which group of animals are more (or less) phenotypically derived on average than other groups (Irie et al., 2018). Echinoderms, for example, are often regarded as “highly derived” species (Hyman, 1955; Morris, 1999; Brusca et al., 2016), as they are a unique group of bilaterians which evolved pentaradial symmetric body plans in adults. However, the idea that echinoderms are highly derived is based on the enumeration of novel traits, such as development of body plan with pentaradial symmetry, and it still remains to be tested if echinoderms indeed experienced greater changes as a whole to their phenotypes (more derived) than their sister groups. Similarly, a detailed examination is needed to determine whether the phylotypic period is the least derived stage during development. Its conservation is often evaluated based on the expression profiles of genes that are shared in all the species (1:1 orthologs), and changes accumulated by non-shared genes are underestimated. To tackle these problems, we propose a transcriptomic “derivedness index” to address the degree of phenotypic changes observed in embryos, inherited from a common ancestor. Specifically, we compared the gene expression profiles of echinoderms (Tu et al., 2012; Li et al., 2018, 2020; Hogan et al., 2020) and chordates (Wang et al., 2013; Hu et al., 2017) to test whether echinoderms are more highly derived than chordates.

## 2 Results

### 2.1 Procedure to Calculate Derivedness Index of Embryonic Transcriptomes

**FIGURE 1:**
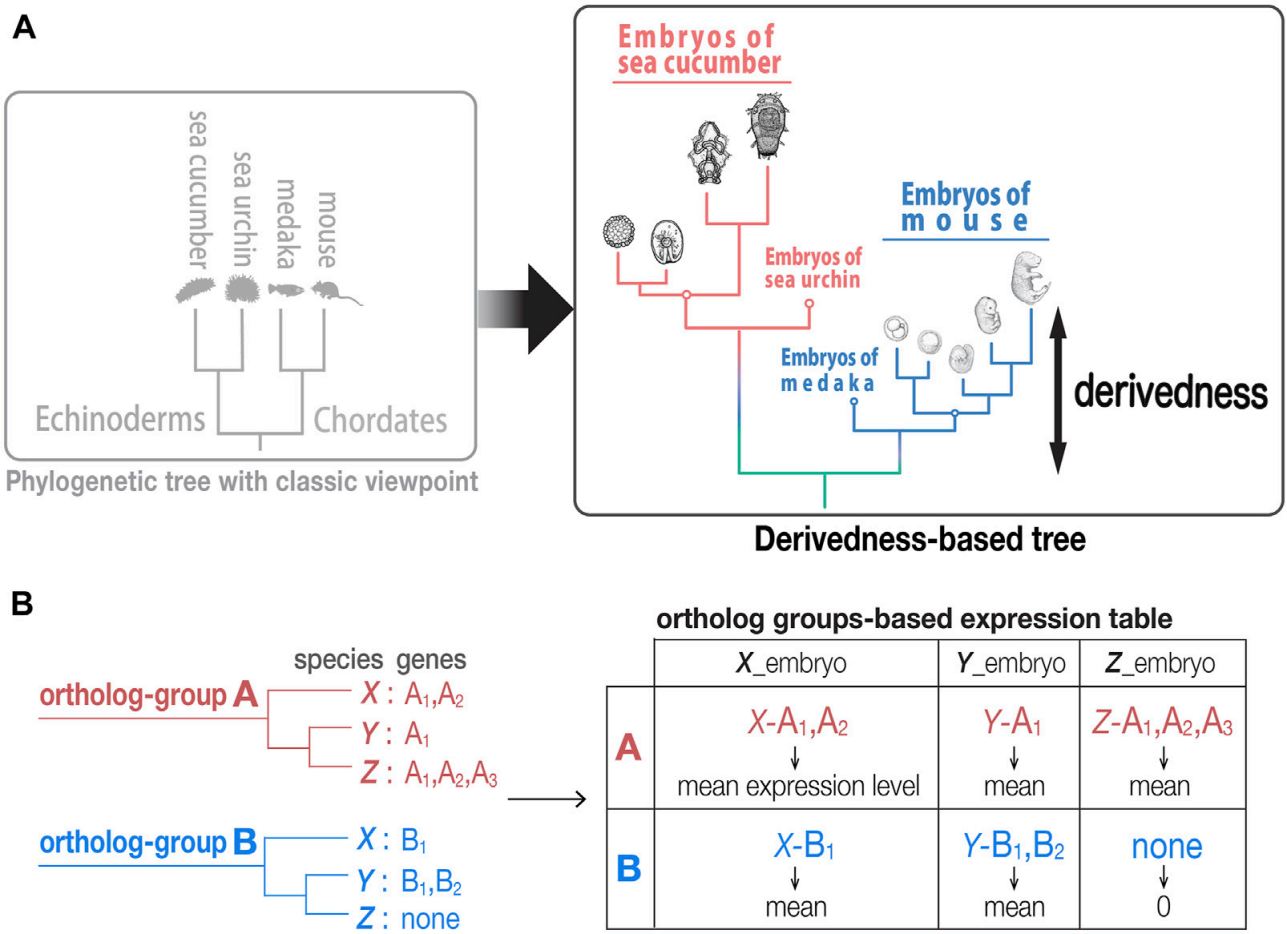
Tree based on phenotypic derivedness of embryos. **(A)** Schematic illustration of a phylogenetic tree drawn by classic viewpoint (left) and that by transcriptomic derivedness (right). Phenotypic derivedness aims to introduce quantitative evaluation of how derived each sample or embryo became from their common ancestor. **(B)** In contrast to evaluating conservation with commonly shared genes, such as 1:1 orthologs, our method takes advantage of ortholog group-based expression table, which considers the expression of paralogs and potential lost genes.

While our recent study indicated that echinoderms have a comparable evolutionary rate in their genomes compared with that of chordates (Li et al., 2020), it still remains to be tested if echinoderms have experienced larger modifications to their development than their sister groups. Meanwhile, it is tempting to know if developmental stages that establish pentaradial symmetry and its later stages are more highly derived than the embryos of their sister groups. To answer this question, we developed a method for quantifying the derivedness of embryos using gene expression profiles (Figure 1A). Specifically, we used whole embryonic developmental transcriptomes as a phenotype of embryos, and compared them between echinoderms (*Anneissia japonica*, *Apostichopus japonicus*, *Lytechinus variegatus*, *Strongylocentrotus purpuratus*) and chordates (*Mus musculus*, *Gallus gallus*, *Pelodiscus sinensis*, *Xenopus laevis*, *Danio rerio*, *Oryzias latipes*, *Ciona intestinalis*, and *Branchiostoma floridae*).

In contrast to approaches that compare the expression of only conserved 1:1 orthologs, our approach encompasses expression of paralogs and potentially lost genes to cover as many evolutionary changes as possible. As indicated in a previous study, 1:1 ortholog-based comparisons encounter a major barrier when multiple species are compared (Hu et al., 2017). In brief, the number of 1:1 orthologs that can be identified comes up to a very small ratio of genes in the entire genome (e.g., only 1,704 1:1 orthologs could be identified in their analysis with eight chordate species (Hu et al., 2017)). This situation is intensified in our analysis when both chordates and echinoderms are involved (13 species in total), where only 271 1:1 orthologs could be identified. These not only account for ∼1% of all genes in a typical vertebrate genome (∼20,000 genes) but also overlook the changes (such as those by gene duplication and/or gene loss) that occur during evolution, which leads to possible underestimation of how derived each embryo is from their common ancestor.

To identify genes in orthologous groups of distantly related species, we first compared protein-coding genes and identified 22,699 ortholog-groups using the PorthoMCL software for eight chordates, four echinoderms, and one outgroup species (Li et al., 2003; Tabari and Su, 2017) (Supplementary Figure S1). We then calculated the normalized expression (see also Table 1 in Methods for the normalization methods tested) for each ortholog-group by 1) taking the mean expression of paralogs and 2) giving “zero” expression value to potentially lost genes (taking sum-expression of paralogs also provided similar results, as in a previous study (Hu et al., 2017); see also Figure 1B and Supplementary Figure S3A). However, species-specific genes were not included, as the analyses including these genes did not meet the criteria we utilized (described in detail below).

**TABLE 1:** Derivedness tree construction method combinations for scanning.

Procedure	Methods to test
Normalization	log_2_ (TPM+1)
	log_10_ (TPM+1)
	quantile normalization
	ascending rank
	descending rank
	z-score
	log_2_ (TPM+1) → quantile
	log_2_ (TPM+1) → z-score
	log_2_ (TPM+1) → quantile → z-score
Distance methods	1—Pearson’s correlation coefficient *r*
	1—Spearman’s correlation coefficient *ρ*
	tEuclidean
	tManhattan
	Cosine
	Canberra
	Jensen-Shannon
Tree inference	NJ (neighbor-joining) (Saitou and Nei, 1987)
	BIONJ (Gascuel, 1997)
	FastME, balanced (Lefort et al., 2015)
	FastME, OLS (Lefort et al., 2015)
	Fitch-Margoliash (PHYLIP) (Fitch and Margoliash, 1967; Revell and Chamberlain, 2014)

**FIGURE 2:**
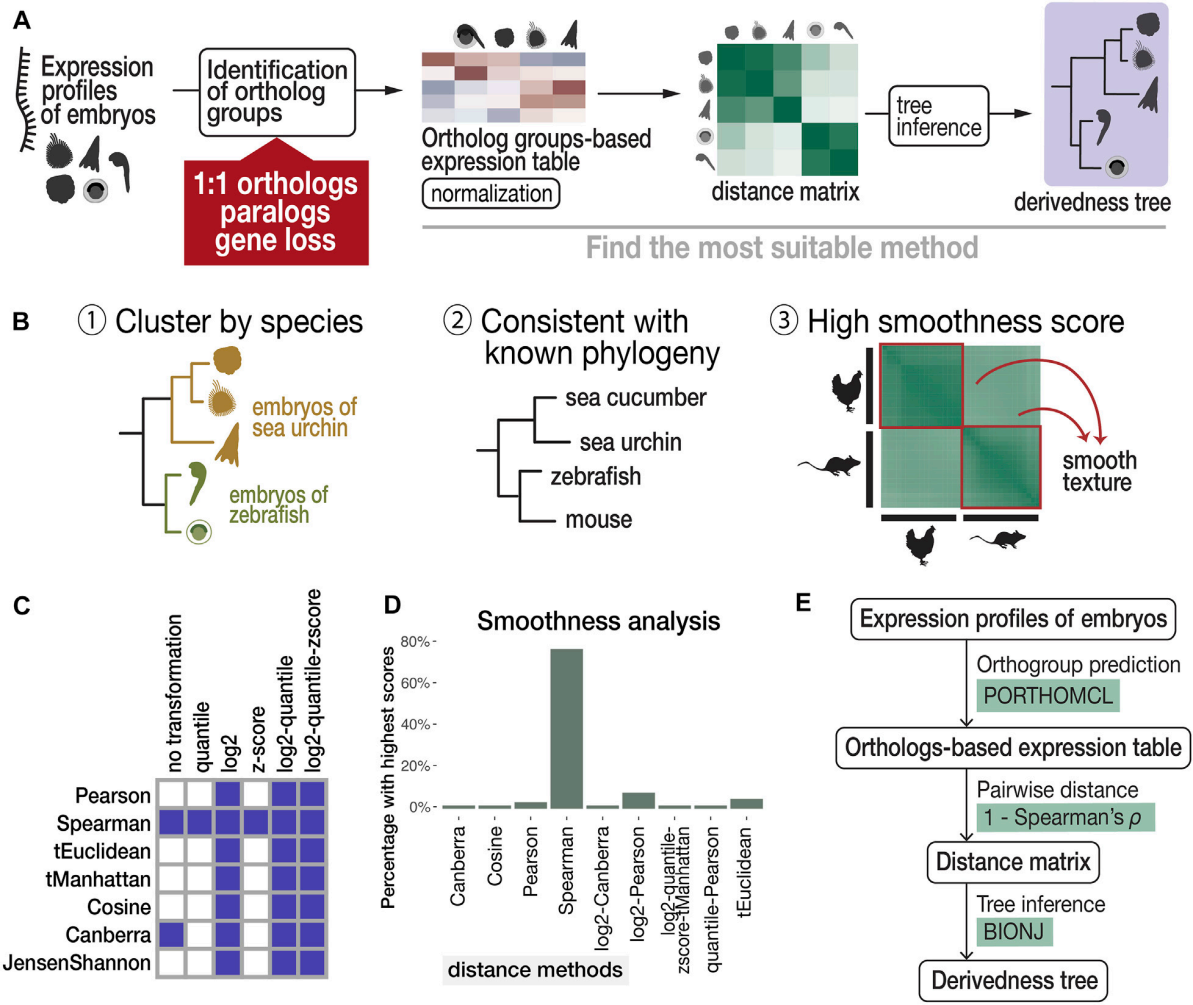
Proposed workflow to estimate phenotypic derivedness of embryos using gene expression profiles and evaluation of methods. **(A)** Outline of measuring transcriptomic derivedness. Whole embryonic transcriptomes from different species were compared using the expression levels of ortholog groups, which consider not only 1:1 orthologs but also paralogs and genes that are presumably lost in specific lineages. Derivedness index of each embryo was defined as the total branch length from the putative common ancestral node on the inferred tree. **(B)** Criteria for selecting the suitable method for quantifying derivedness of embryos. These include: [1] clusters samples by species; [2] topology of derivedness tree consistent with known phylogeny estimated from genomic sequences (Kalinka et al., 2010; Hu et al., 2017), with support from biological replicates; [3] Transcriptomic similarities show gradual change along developmental stages. **(C)** Rank (involved in calculating Spearman distance) and logarithmic normalizations of expression data tend to meet the criteria of clustering stages by species. Shaded boxes represent normalization and distance methods showing this topology in the inferred tree. **(D)** Spearman distance scores the highest in smoothness analysis. (E) Summary of the most suitable method among the combinations tested.

Finally, transcriptomic distance was calculated with various combinations of normalization, distance, and tree inference methods and scanned for a suitable combination (Figure 2A) based on the following three criteria (Figure 2B): 1) developmental stages cluster by species (Kalinka et al., 2010; Hu et al., 2017); 2) tree topology is consistent with known phylogeny inferred from genomic sequences (suggested by previous studies (Kalinka et al., 2010; Hu et al., 2017)); and 3) within-species transcriptomic distances show gradual changes along development (high smoothness in the distance image).

Criterion 1, a topology of developmental stages clustered by species, can be expected for the species covered in this study. For example, even with six closely related *Drosophila* species that split less than 40 million years ago and share very similar morphological features, their whole embryonic transcriptomes still cluster by species rather than by stages (Kalinka et al., 2010). This is presumably because despite sharing many conserved developmental features, larger differences including those with respect to species-specific characteristics exist, such as ovariole number and genome size (Markow and O’Grady, 2007), and this may have clustered samples by species. Development-related genes account for only 10–25% of the whole genome of chordate species (Hu et al., 2017). Therefore, in this study, we selected representative species that should be distantly related enough to avoid the tree topology of clustering by stage. Criterion 2 was based on the assumption that the derivedness of the tree based on overall transcriptomic profiles does not differ from the known phylogenetic topology deduced from the genome, and this is supported by a study that analyzed more closely related *Drosophila* species (Kalinka et al., 2010). For criterion 3, we deployed image texture analysis to score the degree of smoothness of distance matrix images generated by different distance methods, as transcriptomic changes are expected to be continuous along developmental stages.

**FIGURE 3:**
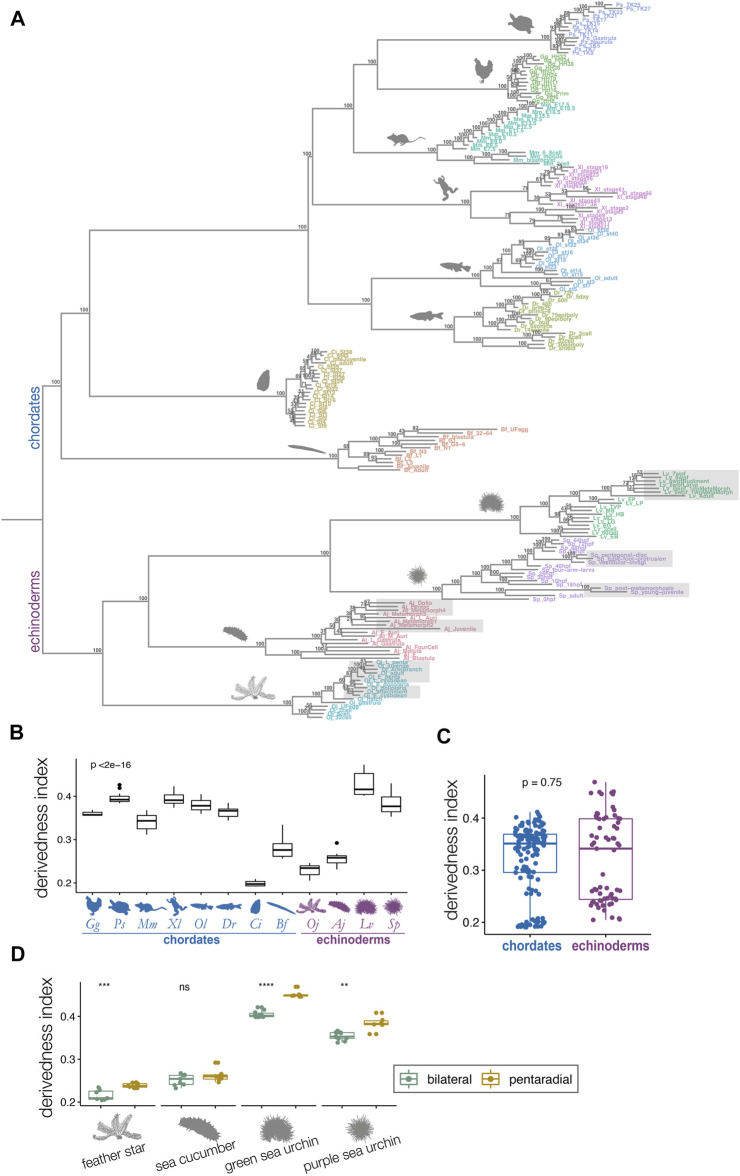
Derivedness tree of developmental stages of echinoderms and chordates based on transcriptomic profiles. **(A)** Tree showing transcriptomic derivedness of chordate and echinoderm embryos. Embryos of each species are denoted by the same color. Support values are consensus from 100 random biological replicates-included (BRI) trees (see also Methods). Shaded stages in echinoderm species are developmental phases with penta-radial structures. **(B)** The range of derivedness indices of embryos for each species. Vertebrate species and the two sea urchin species have fairly high derivedness indices whereas *C. intestinalis* has the lowest (blue: chordate species; purple: echinoderm species; Kruskal-Wallis *p* < 2e-16) **(C)** The range of derivedness indices of echinoderm and chordate embryos. This shows that echinoderms do not appear to have much higher derivedness index than chordates. (Mann-Whitney *p* = 0.75) **(D)** The range of derivedness indices of the bilateral (green) and the pentaradial (yellow) phases of echinoderm development. The penta-radial phase is more derived in feather star (*Oj*), green sea urchin (*Lv*), and purple sea urchin (*Sp*). (Mann-Whitney *U* test, **: *p* < 0.01; ***: *p* < 0.001; ****: *p* < 0.0001; ns: *p* > 0.05). Species abbreviations: *Gg*, chicken; *Ps*, soft-shelled turtle; *Mm*, mouse; *Xl*, clawed frog; *Ol*, medaka; *Dr*, zebrafish; *Ci*, ascidian; *Bf*, amphioxus; *Oj*, feather star; *Aj*, sea cucumber; *Lv*, green sea urchin; *Sp*, purple sea urchin.

The results of our scanning show that both rank and logarithmic transformations of the expression data met criterion 1, namely, clustering samples by species (shown in Figure 2C and in the visualized expression images in Supplementary Figure S2). In addition, we found that calculating pairwise distances of gene expression profiles using “1—Spearman’s correlation coefficient *ρ*” (abbreviated as *Spearman distance* below) meets all three criteria and is the most suitable method among the tested distance methods (Figures 2C, 2D, 3A). Finally, we tested several commonly used tree inference methods, where BIONJ outperformed the other tested methods with its speed and algorithmic design to cluster samples with the lowest distances together (Saitou and Nei, 1987; Gascuel, 1997), although most methods generated similar results except for the Fitch-Margoliash method (Supplementary Figure S3B). A simplified scheme of the most suitable combination based on our criteria is summarized in Figure 2E. As mentioned above, our analyses did not cover the expression of species-specific genes. Intuitively, inclusion of species-specific genes may allow us to more comprehensively cover and evaluate the changes made during evolution; however, we excluded these genes as the analyses including species-specific genes did not meet criterion 2 (consistent with known phylogeny). *X. laevis* became an outgroup of other vertebrates in this tree (Supplementary Figure S4). This may be due to the low accuracy of gene prediction, which led to an excessive number of species-specific genes which biases the distance calculation (Supplementary Figure S1D). Notably, 7,879 (40%) of these non-paralogous, specific-specific genes in *X. laevis* are lowly expressed (max TPM <1) throughout the developmental stages examined, suggesting that many of these genes might be inaccurately annotated.

### 2.2 Penta-Radial Phase of Echinoderms Appear to Be Highly Derived, but Their Overall Developmental Systems May Not Be Much More Derived Than Those of Chordates

We defined the derivedness index of each embryo as the total branch length from the putative common ancestral node on the inferred tree (Figure 3A), which is the common ancestor of chordates and echinoderms. As has been assumed, some of the echinoderm species, especially embryos of two sea urchins, showed much higher derivedness indices than those of invertebrate chordates. Notably, the tendency was more obvious for the developmental stages with pentaradial symmetry (shaded stages in Figure 3A). Meanwhile, the other echinoderms, such as the feather star (an early-diverged echinoderm species (Cannon et al., 2014; Li et al., 2020)) and the sea cucumber showed rather less-derived indices when compared to those of vertebrates (Figure 3A). These tendencies were corroborated by summarizing the range of the derivedness indices of all embryos for each species [Figure 3B; changes in the indices among different species were statistically significant (*p* < 2e-16 by Kruskal-Wallis test)], and that for all chordates and echinoderm species. (Figure 3C). Nevertheless, when we compared the derivedness index of the penta-radial phase of echinoderm development (including metamorphosis, juvenile, and adult stages) against the bilateral phase (early stages to larval stages, before penta-radial structures start to appear) in each species, the penta-radial phase was indeed more derived in the feather star and the two sea urchin species (Figure 3D, statistically significant, tested with the Wilcoxon signed-rank test). Considering that the two sea urchin species, which split around 180 million years ago (Li et al., 2020), share similar developmental characteristics, the reason why *L. variegatus* showed a relatively higher derivedness index than *S. purpuratus* remains to be clarified (*L. varietagus* became even more highly derived when species-specific genes were considered; Supplementary Figure S4). However, this could partially be due to *L. variegatus* having a faster genomic evolutionary rate than *S. purpuratus* (Li et al., 2020; see also Supplementary Figure S19). These results suggest that although echinoderms may not have experienced larger modifications to their molecular developmental systems than chordates since their split from the deuterostome common ancestor, sea urchins appear to have accumulated more changes in their developmental systems, especially in the later embryonic stages when pentaradial structures become evident. However, this may not be solely due to gene expression from pentaradial structures or their source structures, as the sample “Lv_8wpfRudiment” (the forming rudiment in 8-weeks-post-fertilization embryo of *L. variegatus*) is almost as highly derived as “Lv_8wpfLarva” (the remaining larval body with the rudiment removed), which does not contain pentaradial structures. The differences in transcriptomic derivedness indices of the pre-metamorphosis and the pentaradial phases could at least partly be attributed to how genes are expressed at different levels in the two phases (rather than the two developmental phases deploying different sets of genes) (Supplementary Figure S17). Further studies delineating the molecular mechanisms of the metamorphosis process may help explain why stages with pentaradial structures tend to show highly derived indices.

### 2.3 “Conserved” Phylotypic Periods Are the Least Derived in Vertebrates

**FIGURE 4:**
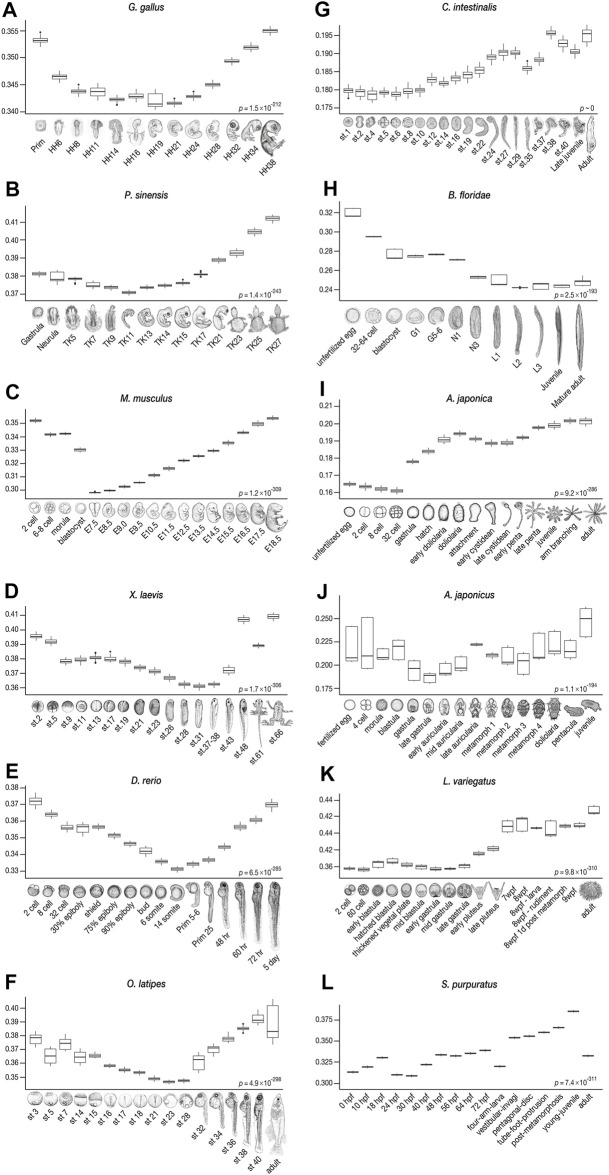
Derivedness index of embryos of chordates and echinoderms estimated from their respective common ancestors. The range of derivedness indices of each embryo, from the common ancestor of chordates and echinoderms, respectively, in 100 biological replicates-included (BRI) trees. **(A–F)** The least derived stages in vertebrate species (*Gg*, *Ps*, *Mm*, *Xl*, *Dr*, and *Ol*) are mid-embryonic and organogenesis stages (*Gg*: HH14-19, *Ps*: TK11, *Mm*: E7.5*, *Xl*: stage 31, *Dr*: 14-somites, and *Ol*: stage 23). *: E9.5, in mouse when the Quartz-Seq samples were removed (see Figures 6B–6ii). **(G–H)** The tunicate *C. intestinalis* shows relatively lower derivedness indices in stage 1 to stage 10 embryos, and the least derived stage in the amphioxus *B. floridae* is around the L2 (open-mouth larva) stage. **(I–L)** In echinoderm species, the least derived stage is around the gastrula in sea cucumber and sea urchins, whereas the 32-cell stage is the least derived in feather star. (Differences in derivedness index for each developmental stage are statistically significant; Friedman test).

Consistent with previous studies that supported the developmental hourglass model with 1:1 orthologs (Irie and Kuratani, 2011; Levin et al., 2012; Wang et al., 2013; Uesaka et al., 2019), our results show a similar trend in vertebrates, with conserved mid-embryogenesis (especially organogenesis stages) being the least derived within each species from the common ancestor of chordates (Figures 4A–F). Meanwhile, in some species, the identified least derived stages showed a slight shift from the previously reported conserved stages; for instance, while Prim-5-6 of the zebrafish (*D. rerio*) was previously suggested to be the most conserved stage (or vertebrate-phylotypic period), but the least derived stage was at a slightly earlier, 14-somites stage, and the Prim-5-6 stage was the second least derived embryo (see also Supplementary Figure S7). Larger differences were observed in *Ciona* and amphioxus. While the most conserved stages, identified by 1:1 orthologs, in *Ciona* were around st.24–29, the least derived stages were st.1–10. In amphioxus, while the mid-neurula stage (Hu et al., 2017; Marlétaz et al., 2018) was the most conserved, stage L2 (open-mouth larva) was found to be the least derived. These results imply that “conserved” phylotypic period are in general less derived than the earlier/later stages, but these may show larger differences especially when more number and/or distantly related species are being compared.

Nevertheless, among the species analyzed, for the range of derivedness indices and position of the common ancestral node of all the embryos of each species, the Chinese soft-shell turtle tends to show more derived developmental systems than the other vertebrates. This is consistent with the view that turtles possess many highly derived morphological diapsid features (Nagashima et al., 2009; Gilbert and Corfe, 2013). However, genomic analyses indicated that turtles have a slow evolutionary rate (Bradley Shaffer et al., 2013; Green et al., 2014), and further studies are required to explain this discrepancy. Meanwhile, given that the fossil records of turtles remained morphologically conserved since their appearance (Li et al., 2008; Benton, 2014), it is possible that the turtle embryos are transcriptomically more derived (expressing orthologous genes in a different way) than the other vertebrate species while maintaining a slow genomic evolutionary rate. In contrast, our results indicate that the mouse appears to possess the least derived molecular developmental system among the vertebrates compared (Figures 3B, 6B-iii). Another unexpected result was that the overall stages of *Ciona* showed the least derived indices when compared with those of the embryos of other chordates, including the early diverged amphioxus. This is in contrast to both morphological and genomic studies, which suggested that tunicates could be the most derived species in chordates (Holland, 2015, 2016). Moreover, a similar tendency was also corroborated by the tree that covered species-specific genes (Supplementary Figure S4) and the tree drawn by 1:1 orthologs (Supplementary Figure S5). The exact reason for this is unclear; however, a potential reason would be due to the vast number of potentially lost genes in *Ciona*, as it may bias the distances against different species by giving higher transcriptomic similarity to the rest of the species. Given that our method correctly captured transcriptomic derivedness, the results imply that their orthologous genes used during embryonic development remain rather ancestral, despite their highly divergent genomic sequences (Berná and Alvarez-Valin, 2014) after the split from the Olfactores common ancestor.

### 2.4 Characterization of Least Derived Stages by Extracting Highly Expressed Ortholog-Groups

**FIGURE 5:**
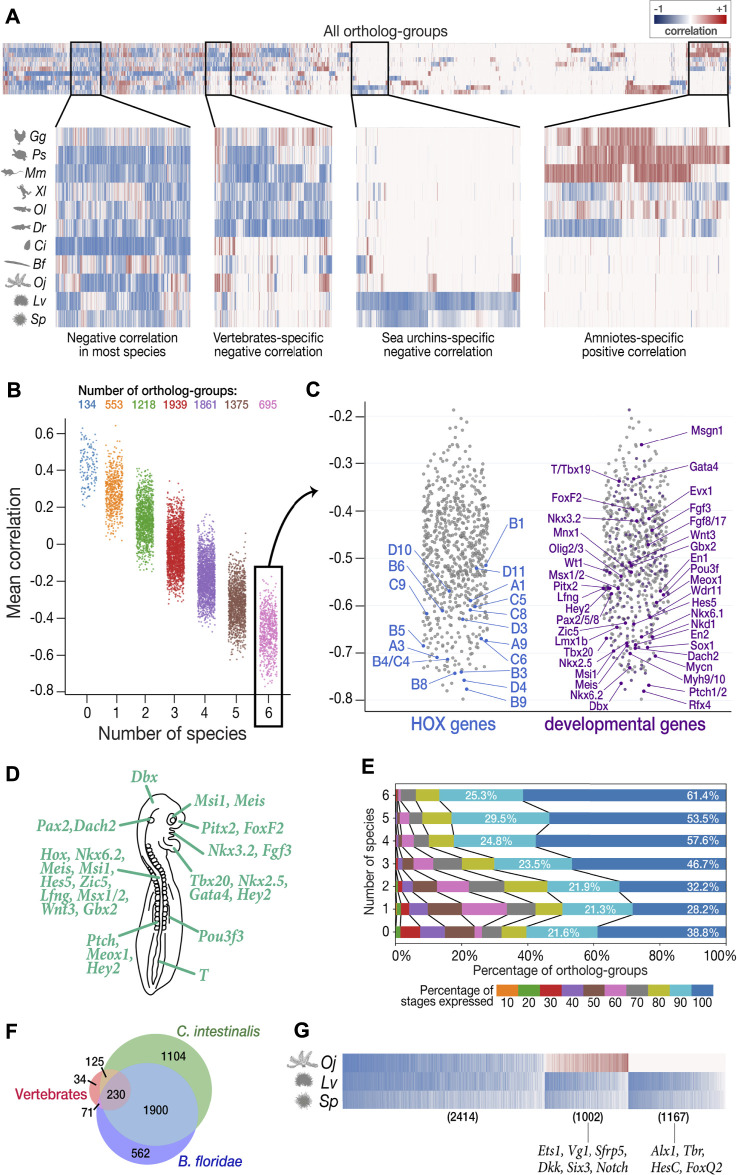
Characterization of least derived stages by ortholog-groups. **(A)** Heatmap showing Spearman’s correlation coefficient between expression levels and derivedness indices for each ortholog-group for each species (blue: negative correlation; red: positive correlation). Selected areas in the heatmap are zoomed in to show clusters of ortholog-groups with (from left to right) negative correlation in most species; negative correlation in most vertebrate species; negative correlation only in the two sea urchins; positive correlation in amniotes (mouse, chicken, and turtle). **(B)** Ortholog-groups showing negative correlation (negative DCOs) in vertebrates. 7,775 ortholog-groups were analyzed for vertebrates, and were further classified into seven categories based on the number of species in which they show negative correlation. 695 ortholog-groups in category 6 (showing negative correlations in all six vertebrate species analyzed) were further analyzed in **(C–E)**. **(C)** Category 6 negative DCOs, including 18 HOX ortholog-groups (left) and 201 development-related ortholog-groups (right). **(D)** The putative phenotype of the least derived/conserved mid-embryonic stage of vertebrates. Genes known to express in homologous anatomical structures during this developmental phase in mouse, chicken, frog and zebrafish are highlighted. **(E)** DCOs with negative correlations in more species tended to show higher degree of temporal pleiotropy, estimated by the ratios of stages detected (TPM ≥ 1). **(F)** The number of DCOs with negative correlations in 6 vertebrate species (red), *C. intestinalis* (tunicate; green), and *B. floridae* (amphioxus; blue). 230 ortholog-groups showed negative correlations in all three groups, suggesting that they might be involved in ancestral functions retained in chordates. **(G)** 2,414 negative DCOs were detected across echinoderm species (left); however, the functions of most of these genes remain unknown. Well-studied gastrulation-related genes, such as *Ets1* and *HesC*, were among the groups showing positive correlation in feather star (middle) and the sea urchin-specific gene group (right), respectively.

While there were a few reports that hint how the body plan-establishing phase became conserved (Bogdanović et al., 2016; Hu et al., 2017; Zalts and Yanai, 2017; Uchida et al., 2018), we further sought for potential hints toward the mid-embryonic conservation. Specifically, we asked which ortholog-groups could potentially characterize this conserved phase, given that the least derived stage only differed slightly from the reported conserved phase in vertebrates. For this purpose, we sought ortholog-groups that were highly/lowly expressed during the stages with less derivedness, by calculating the correlation (Spearman’s correlation coefficient) between the expression of each ortholog-group and derivedness indices during development. For example, ortholog-groups with strong negative correlations across species (dark blue in Figure 5A, abbreviated as “negative DCOs,” derivedness-correlative ortholog-groups) tended to show higher expression around the less derived developmental phase and lower expressions in more derived stages. We especially looked for negative DCOs across the six vertebrate species, as they may represent transcriptomic features of the phylotypic period and could potentially provide a hint about the mechanism of its conservation.

To avoid unexpected bias, we excluded samples obtained by Quartz-Seq (2-cells to blastocyst in mouse and all stages in sea cucumber) for this analysis, as these were obtained by different RNA-seq protocols than the others (discussed in detail in the next section). Among the 22,699 ortholog-groups analyzed, we focused on 7,775 ortholog-groups that had at least one gene counterpart in each vertebrate species (Figure 5B). Among these, we found 695 ortholog-groups showing negative correlation in all six vertebrate species concomitantly (category 6 in Figure 5B; Supplementary Table S19), including 18 HOX ortholog-groups, 201 development-related ortholog-groups, and 161 ortholog-groups involved in signaling transduction (Figure 5C, Supplementary Figures S8–10; the ratio of development-related ortholog-groups is significantly higher in category 6 than in any other category; Fisher’s exact test), consistent with the actively proceeding organogenesis in these stages. Along with whole embryonic expression, some of aforementioned genes are also known to show conserved spatial expression patterns (Thisse et al., 2004; Darnell et al., 2007; Yokoyama et al., 2009; Bowes et al., 2010; Richardson et al., 2014; Smith et al., 2018) (Figure 5C). Of note, these negative DCO genes that show spatial conservation contain many genes that are reported to be involved in neural patterning. For example, *Nkx6.1*, *Pax2*, *Lmx1b*, *Wt1*, *Evx1/2*, *En1*, *Mnx1*, *Sox1*, and *Olig2/3* are involved in the dorsal-ventral patterning of neurons in the spinal cord (Catela et al., 2014). Additional examples are the two ortholog-groups with the strongest mean negative correlation. *Patched1/2* and *Rfx4*, which are receptors of sonic hedgehog (Shh) and a downstream target of Shh signaling, respectively, whose mutants show severe defects in the neural tube (Goodrich et al., 1999; Ashique et al., 2009; Murdoch and Copp, 2010; Sedykh et al., 2018) (see also Supplementary Figure S11; Shh also showed negative correlation across species). Genes involved in other conserved structures of the phylotypic embryo were also identified as negative DCOs (Figures 5C,D), such as those involved in somitogenesis (*Patched1/2*, *Nkd2*), heart (*Nkx2.5*, *Tbx20*, *Gata4*), and mesonephros (*Pou3f3*). Besides, this list of negative DCOs also included genes that were not previously considered to be directly related to embryonic development, such as *Fidgetin* (the ortholog-group showing the strongest mean negative correlation of −0.798 among all ortholog-groups analyzed). *Fidgetin* has been recently suggested to regulate the cytoskeleton in the spinal cord and somites in the mouse embryo (Leo et al., 2015). More genes that are involved in organogenesis could potentially be identified from these DCOs.

In addition, consistent with a recent report suggesting that developmental stages with more pleiotropic (repetitively used) genes tend to be evolutionarily conserved, ortholog-groups showing negative correlation in more species tended to be expressed in more developmental stages (temporal pleiotropy, Figure 5E), suggesting that negative DCOs are potentially useful for characterizing the least derived stages. However, we note that this method may overlook ortholog-groups that have significant contribution to the phenotype of the least derived stages but show consistently high expression throughout development because they may not show a strong negative correlation with the derivedness index.

Finally, we extended the analysis to the entire chordate and echinoderm clades, which have been difficult to perform with 1:1 ortholog-based or conservation-oriented methods (as only ∼1% protein-coding genes of the entire genome could be compared). Among the 4,026 chordate ortholog-groups, 230 were identified as negative DCOs in all eight chordate species analyzed (Figure 5F, Supplementary Table S20). As expected, these contained a variety of genes that are known to be important for chordate embryogenesis, such as *Tbx20* (Belgacem et al., 2011), which showed a strong average negative correlation. This implies that negative DCOs might provide hints for the identification of genes retaining conserved functions shared in chordates in future studies, however, it also has to be noted that sequence-based similarity does not always indicate their similar functions, and roles of negative DCOs have to be analyzed carefully in the future studies. Similarly, in echinoderms, we found 2,414 DCOs showing negative correlation across all three echinoderm species (left-most in Figure 5G, Supplementary Table S21), which contained genes mainly expressed in gastrula in sea urchins and 2-cell to 32-cell stage in feature star (the least derived stages shown in Figure 4I–L). Since the functions of genes in echinoderm species remain largely unexplored, we could not deduce much information from the negative DCOs in echinoderms; however, some implications were obtained for smaller groups in echinoderms, namely, sea urchins (Figure 5G). In contrast, genes involved in the establishment and growth of the pentaradial phase were included in the cluster showing positive correlation in all three echinoderm species since this developmental phase was shown to be more derived.

### 2.5 Technical Concerns in Cross-Species Transcriptomic Analyses

**FIGURE 6:**
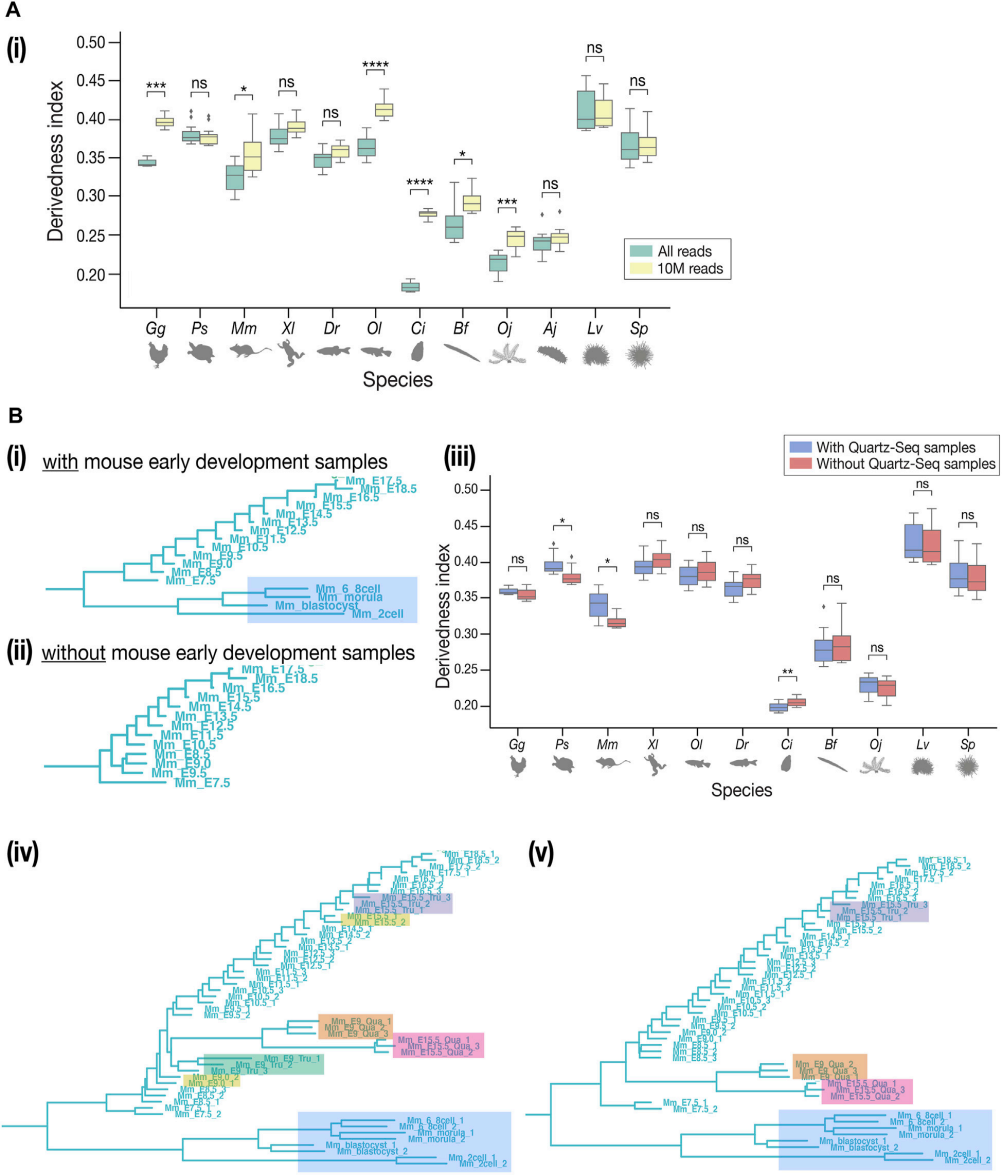
Technical concerns in derivedness tree inference. **(A)** Read depth control. (i) In the tree inferred from 10 M-controlled expression data, the range of derivedness indices significantly increased in some species (*Gg*, *Mm*, *Ol*, *Ci*, *Bf*, and *Oj*). **(B)** Comparison of TruSeq and Quartz-Seq datasets. **(i)** The samples of early development in mouse (shaded in blue) and all samples of sea cucumber (not shown) were collected by Quartz-seq. **(ii)** Removing the Quartz-seq samples changed the tree topology inside the mouse clade. The least derived stage became E9.5. **(iii)** After removing the Quartz-seq samples, the range of derivedness indices for most species were not greatly affected, not influencing the conclusions we drew in the previous sessions. **(iv–v)** Tree with all original samples and the new samples to compare TruSeq & Quartz-seq datasets. Unexpectedly, the samples tend to cluster by protocols rather than by stages or biological replicates. This tendency is stronger in (v) where the E9.0 TruSeq samples were omitted, while the E15.5 Quartz-seq and Tru-Seq samples still did not cluster together. Samples from the same starting total RNA but by different protocols (such as E15.5_Tru_1 and E15.5_Qua_1) did not cluster together either. (Shaded in orange: E9.0 by Quartz-seq; green: E9.0 by TruSeq; pink: E15.5 by Quartz-seq; purple: E15.5 by TruSeq; yellow: E9.0 and E15.5 by TruSeq from the published dataset). For **(A-i)**, **(A-ii)**, and **(B-iii)**, Mann–Whitney–Wilcoxon test was performed (*: *p* < 0.05; **: *p* < 0.01; ***: *p* < 0.001; ****: *p* < 0.0001; ns: *p* > 0.05).

While many of our results correspond with previous EvoDevo studies, it has to be noted that there are several technical issues to overcome. First, the choice of outgroup species may affect the topology of the tree, as is the case in most evolutionary studies. In contrast to Figure 3A, the tree with *Drosophila melanogaster* as the outgroup (Supplementary Figure S13) showed a tree topology that deviated more from the known phylogeny. This could potentially be because fewer genes in *D. melanogaster,* (which is often considered a highly derived species in arthropods (Andrioli, 2012), could be identified as orthologous genes of deuterostomes. Second, differences in read depth may cause biases in expression quantification, as samples with deeper sequencing are expected to detect more lowly expressed genes than shallower samples. To test how these may affect the derivedness index, additional trees were plotted from expression data with depth adjusted uniformly to 10 million (randomly picked up 10 million mapped reads, 10 M; which corresponds to the depth of the shallowest sample), gradual down-sampling of read depths, or adjusted proportionally to the exome size of each species. In the tree with the 10 M expression data, the range of derivedness indices for six species, notably *Ciona*, increased significantly (Figures 6A–i, see also Supplementary Figures S14, S16), while the tree with exome sizes-adjusted depths showed significant changes in fewer species (Supplementary Figure S15). Notably, the topologies of both trees were inconsistent with known phylogeny. This suggests that although the derivedness index tends to be influenced by read depth, the influence may be dependent on the species or the samples; thus, more comprehensive studies are needed to determine which read depth normalization method would be more suitable for measuring transcriptomic derivedness. Finally, as we included samples acquired by different library preparation protocols where the Quartz-Seq protocol involved an additional whole-transcript amplification step (Sasagawa et al., 2013), we asked whether these samples were comparable to each other. To answer this, we collected new RNA-seq data from E9.0 and E15.5 mouse embryos by performing both TruSeq and Quartz-Seq from the same starting total RNA sample. A tree with all samples, including biological replicates, was plotted. Unexpectedly, samples tended to cluster by protocols rather than by stages or biological replicates (Figures 6B-iv,v), suggesting that samples obtained by these two protocols may not be completely comparable. To avoid this bias, we excluded samples acquired by Quartz-Seq for the analysis of derivedness-correlative ortholog-groups.

## 3 Discussion

The quantitative concept of the degree of phenotypic evolution, or phenotypic derivedness, originates from the early history of evolutionary thoughts when taxonomists attempted to compare degree of evolution of traits (Mayr, 1982), but further development of methods under modern evolutionary theory was mostly not pursued. This could partly be owing to multiple factors or understandings, such as *scala naturae* of the pre-Darwinian era (Diogo et al., 2015) and Haeckel’s recapitulation theory [reviewed in (Kuratani et al., 2021)]. We contend that rather than simply abrogating this idea or mixing it with the concept of conservation, the quantitative degree of phenotypic evolution, or derivedness, may help us more deeply understand phenotypic evolution. As an application, we developed a transcriptomic derivedness index that considers not only the expression levels of strictly conserved 1:1 orthologs, but also those of paralogs and potentially lost genes. This contrasts with previous approaches, which only focused on strictly conserved 1:1 orthologs, whereas our method covers most of the genes in the genome of each species. While species-specific genes could not be included since the analysis including these genes violated one of the criteria set for this study, our method is still advantageous than the 1:1 orthologs-based method. This is because the differences in the genes being covered for each species become extremely small in the previous method, especially when a large number of species are being compared. Our scanning demonstrated that calculating evolutionary distance between embryonic transcriptomes using Spearman distance meets the criteria, including phylogenetic topology estimated by transcriptomic information recapitulates that estimated by genomic information, and the estimated tree topology show “developmental stages cluster by species”.

Using this transcriptomic “derivedness index,” we quantified the derivedness of whole embryonic transcriptomes by utilizing gene expression profiles of echinoderm and chordate embryos, and tested whether echinoderms are highly derived species (Hyman, 1955; Morris, 1999; Brusca et al., 2016). Unexpectedly, the tree (Figure 3) suggested that developmental systems of echinoderms might not have experienced larger modifications than those of chordates since the split from their common ancestor, the penta-radial phase of echinoderm species tends to be highly derived, as had been assumed. Meanwhile, in the vertebrate clade, we found that conserved mid-embryonic stages (the phylotypic period) in vertebrates tended to be less derived as well (Figure 4). In contrast to the situation in vertebrates, larger differences between the least derived stage and the most “conserved” stage were observed in the tunicate *C. intestinalis* and the amphioxus *B. floridae*; however, the reason is unclear*.* The least derived stages in *C. intestinalis* (cleavage) do not seem to span developmental phases responsible for the conserved anatomical structures of chordates [including notochord, pharyngeal gill slits, dorsal nerve cord, and segmental muscles (Benton, 2014; Holland, 2015). In contrast, in amphioxus, the larval stages with developing gill slits were identified as the least derived. Hence, the least derived stages across the chordates phylum may not be the developmental phase involving patterning of chordate-specific and conserved anatomical structures, as has been implied by previous studies (Holland, 2015; Hu et al., 2017). Alternatively, it is also possible that the phylotypic period of *C. intestinalis* became highly diversified. However, a caveat of our results would be that it may not be suitable to infer relationships between phylogenetic groups and developmental stages [such as which phylogenetic group of animals follow the hourglass model, or to find recapitulative tendencies during development (Kuratani et al., 2021; Uesaka et al., 2021)].

We also pinpointed the turtle showing high transcriptomic derivedness, which is consistent with the morphological perspective, but the result is contrary to those of genomic studies that indicated a slow genomic evolutionary rate (Bradley Shaffer et al., 2013; Green et al., 2014). An even more unexpected finding was that tunicate *C. intestinalis* showed the lowest transcriptomic derivedness indices among all the chordate species compared with recent genomic and morphological studies suggesting that tunicates could be a group of rapid-evolving species (Berná and Alvarez-Valin, 2014; Holland, 2016). Transcriptomic datasets of additional tunicate species may help confirm whether this trend is general to tunicates or specific to *C. intestinalis*. Given that our approach accurately captured transcriptomic derivedness, these results suggest that high transcriptomic derivedness may not directly reflect high evolutionary rates in their genomes inferred from commonly shared, conserved sequences.

Nonetheless, these apparent contradictions highlighted excellent opportunities to further revise and improve our method for evaluating the derivedness of embryos in future studies. Ideally speaking, although derivedness should be evaluated together with species-specific genes, the tree estimated using our method with species-specific genes violated the criterion to show topology consistent with known phylogeny (Supplementary Figure S4). This implies that either better criteria for tree topology should be invented or better models of transcriptome evolution should be incorporated when calculating the evolutionary distance between embryonic transcriptomes. Indeed, it could even be possible that transcriptomic derivedness may not perfectly match with phylogenetic relationship deduced from genomic information, breaking the criterion 2 (derivedness tree follows the known phylogeny deduced from genomic information). For example, phylogenetic trees based on genomic sequences mainly rely on those alignable between different species, resulting in exclusion of species-specific genomic sequences. On the other hand, our methodology included information (gene expression levels) that are potentially lost in certain species. These differences could lead to inconsistencies in their phylogenetic tree topologies. In addition, another possibility would be that species having very different evolutionary speed in their phenotypes (including their developmental transcriptome) and genomic mutational rate could also lead to inconsistent phylogenetic results when compared with other species. Second, we proposed assigning “0” expression levels to potentially lost genes. However, in addition to the technical difficulty of definitively identifying lost genes, it also remains unclear how these “0”-expressing ortholog-groups would affect the calculated transcriptomic derivedness, especially species that lost many genes, such as *C. intestinalis*. In addition, further studies are needed to understand how bias can be minimized from other technical aspects, including read depth. For instance, including closely related species, or even populations in the same species, would offer a way to measure technical biases, as these are expected to have similar transcriptomic profiles. Our method could also be potentially biased by the differences in genome annotation quality among species; however, since annotation quality depends on a variety of factors, such as how many genome-sequenced closely related species are available and sequencers or assembly methods used for each species, it is unfeasible, at least at this moment, to adjust the quality of annotations in different species. Importantly, to obtain a more comprehensive picture of the derivedness of embryos, transcriptomes should not be the only parameter to measure; instead, other aspects such as epigenomes, morphologies, and changes observed in fossil records, and cell types could also contain a lot of information about derivedness and should be considered.

Finally, we attempted to characterize the least derived developmental stages of vertebrate species by extracting ortholog-groups showing a negative correlation between the derivedness index and expression levels during development. Remarkably, the extracted orthologous gene set included numerous genes known to be expressed in the shared anatomical structures of vertebrates during this period. Given that the least derived stage may represent a period with the inclination to retain the ancestral phenotype, in line with the recent perspectives that suggested the constraints in this least derived/conserved period may contribute to the strict conservation of animal body plans (Hu et al., 2017; Furusawa and Irie, 2020), this set of orthologous genes (negative DCOs across vertebrate species) may provide additional insights into the evolutionary mechanisms behind the conservative features of body plans. Taken together, further development of the derivedness index could be a useful quantitative indicator to further study which developmental processes are potentially less evolvable [including those argued in line with developmental constraints (Smith et al., 1985; Galis, 1999; Irie and Kuratani, 2014; Hu et al., 2017; Furusawa and Irie, 2020) and developmental burden (Riedl, 1978; Fujimoto et al., 2021)].

## 4 Methods

### 4.1 Animal Use and Care

Experimental procedures and animal care were conducted in strict accordance with the guidelines approved by the University of Tokyo (approval ID: 14-03, 16-2). The animals were sacrificed with minimal suffering. Individual embryos were blindly selected from the wild-type population.

### 4.2 Embryo Collection, RNA Extraction, Library Preparation, and RNA-Seq

The RNA-seq data utilized were from published datasets (Wang et al., 2013; Hu et al., 2017; Ichikawa et al., 2017; Li et al., 2018, 2020) and three other studies [purple sea urchin (Tu et al., 2012); oyster: (Zhang et al., 2012); *Drosophila*: (Nègre et al., 2011):]. These include major early to-late developmental stages of eight representative chordate species (*Mus musculus*, *Gallus gallus*, *Pelodiscus sinensis*, *Xenopus laevis*, *Danio rerio*, *Oryzias latipes*, *Ciona intestinalis*, *Branchiostoma floridae*), four echinoderms (*Anneissia japonica*, *Apostichopus japonicus*, *Lytechinus variegatus*, and *Strongylocentrotus purpuratus*), and two outgroup species (*Crassostrea gigas*, *Drosophila melanogaster*), with 2-3 biologically independent replicates of each stage to represent the general population (except *S. purpuratus*, *C. gigas*, and *D. melanogaster*). All samples were sequenced using Illumina platforms. Details of the included datasets are summarized in Supplementary Tables S1–13 (developmental stages covered) and Supplementary Table S14 (e.g., sample accession numbers, sequencing platforms).

To compare datasets collected by TruSeq and Quartz-Seq (Sasagawa et al., 2013) protocols (the latter involves an additional whole-transcript amplification step), additional samples of mouse E9.0 and E15.5 (C57BL/6J strain, CLEA Japan) were prepared following the same procedures reported in the mouse and sea cucumber datasets (Hu et al., 2017; Li et al., 2018). Both TruSeq and Quartz-seq were performed on the same total RNA extracted from pooled embryos. An average of nine embryos from three independent parents were pooled for each E9.0 sample, and an average of three embryos, E15.0. Library preparation for sequencing (TruSeq: single-end, 100 bp, non-strand-specific; Quartz-Seq: paired-end, 100 bp, non-strand-specific) was performed according to the manufacturer’s instructions using the Illumina TruSeq RNA Library Prep Kit v2 or the standard protocol of Quartz-Seq (Sasagawa et al., 2013) with Illumina Nextera XT Library Prep Kit. The normalized libraries were sequenced using an Illumina HiSeq 1,500. The raw reads data are available in SRA through the accession number PRJNA749373.

### 4.3 RNA-Seq Mapping

Adapter sequence trimming and low-quality read filtering in RNA-seq raw reads were performed with trimmomatic (version 0.36) with default parameters (ILLUMINACLIP:2:30:10, LEADING:3, TRAILING:3, SLIDINGWINDOW:4:15, and MINLEN:36) (Bolger et al., 2014). Quality checks were performed using FastQC (https://www.bioinformatics.babraham.ac.uk/projects/fastqc/). Reads were aligned to the respective genome using HISAT2 (version 2.1.0) (Kim et al., 2019), with mitochondrial scaffolds and NUMT sequences manually removed from the genomic sequence files to avoid multi-copy quantification biases. The genome versions are summarized in Supplementary Table S15. The expression levels of coding genes were quantified in TPM (transcripts per million) (Wagner et al., 2012) using StringTie (version 1.3.4d) (Pertea et al., 2015).

### 4.4 Construction of Ortholog-Group-Based Expression Tables

Gene-based expression profiles of embryos of all species were summarized into an ortholog-group-based expression table by the method explained in the main text and Figure 1B. Our method was modified from the approach previously reported by the EXPANDE Project Consortium (Hu et al., 2017). Genes were first grouped into ortholog-groups using PORTHOMCL (Li et al., 2003; Tabari and Su, 2017) with a BLASTP (version 2.7.1) e-value threshold of 1e-5, as an approach considering paralogs and potentially lost genes. The expression level per ortholog-group was then estimated by the mean (or sum) expression level of all genes in the ortholog-group in each species. The expression level of “0” was assigned to an ortholog-group with no predicted gene in that species.

### 4.5 Scanning of Methods for the Construction of Derivedness Tree

#### 4.5.1 Derivedness Tree Construction

The derivation of the derivedness tree was based on a pairwise evaluation of transcriptomic similarities between embryos. The tree was inferred from the calculated distance matrix based on the ortholog-group-based expression table (Figure 2A). The most suitable method was searched through combinations of major methods for expression data normalization, distance calculation, and tree inference (Table 1) to meet the three criteria discussed in the main text (Figure 2B). In expression data normalization, log (TPM+1) was used to avoid the undefined value of “log0.” The trees were plotted using the “ggtree” package in R (Yu et al., 2016).

#### 4.5.2 Biological Replicates of Expression Data

For each developmental stage, the expression level of a gene can be represented by the mean value of its expression levels in all biological replicates. These mean-value expression data were used to generate the tree shown in Figure 3A, and all other trees in the Supplementary Material, if not further specified. However, this method may incorporate false-positives and false-negatives in individual sample sets. To avoid this potential bias, we incorporated deviations of gene expression levels in different biological replicates and created “biological replicates-included (BRI)” expression data: a set of BRI expression data randomly takes one biological-replicate sample for each developmental stage. As such, many combinations can be acquired (for example, three biological replicates for 10 developmental stages each can create 310 different combinations) to simulate expression changes during early-to-late development. Support values on the tree in Figure 3A were calculated as the consensus of trees inferred from 100 random BRI expression tables, and these values represent the strength of the biological replicates supporting the tree topology. For the trees inferred from Spearman distance, the consensus tree of 100 BRI trees showed a topology similar to that inferred from the mean-value expression data.

#### 4.5.3 Smoothness Analysis

As gene expression profiles are expected to show gradual changes during development, this is reflected in the distance matrix showing a smooth texture. We adapted texture analysis in image processing theory (Materka and Strzelecki, 1998; Gonzalez and Woods, 2007) to investigate which distance method generated the smoothest matrix image in the within-species comparison regions (Figures 2B–3–3). The smoothness of each within-species comparison region on the distance matrix image was measured using the following descriptor statistics: homogeneity, dissimilarity, contrast, uniformity, and correlation (Supplementary Table S16), with a sliding window of 3 × 3 using the GLCM package in Python. For each within-species region on the distance matrix image, the distance method that scored the best according to each descriptor was recorded, and the percentage of each distance method being selected as the best-scoring method is plotted in Figure 2D.

### 4.6 Analyses Using Derivedness Index

The branch length of each embryo from the common ancestral node on the tree (putative common ancestor of chordates and echinoderms) was extracted and defined as the derivedness index. In calculating derivedness indices for species as a whole (for whole life cycles), the range of derivedness indices of all the developmental stages in the inferred tree (Figure 3A) was represented as box plots (whether the differences among species were statistically significant were examined by Kruskal-Wallis test; Figure 3B). To compare the derivedness indices of penta-radial phase against pre-metamorphosis stages in each echinoderm species (Figure 3D), the range of the derivedness indices of the corresponding developmental phase was represented as a box plot (Mann-Whitney *U* test). Penta-radial phase for each species was defined as: (feather star) doliolaria onwards; (sea cucumber) metamorph-1 onwards; (green sea urchin) 7wpf onwards; (purple sea urchin) vestibular-invagi onwards. To examine the derivedness index of each developmental stage (Figure 4), the results from the 100 random BRI trees were utilized, and for each developmental stage, the range of derivedness indices in the 100 trees was summarized for each embryo (whether the differences among developmental stages were statistically significant were examined by Friedman test).

### 4.7 Identification of Derivedness-Correlative Ortholog-Groups

Samples collected by Quartz-Seq were omitted from the analysis to avoid any unexpected bias. For each species and ortholog-group, Spearman’s correlation coefficient between its expression levels and derivedness indices along early to late embryos was calculated. Ortholog-groups showing negative correlations across certain groups of animals (vertebrates, chordates, and echinoderms) were further analyzed. A total of 7,775 DCOs, with at least one gene counterpart in each vertebrate species, were classified into seven categories based on the number of vertebrate species that showed a negative correlation.

#### 4.7.1 Gene Name and Functional Prediction of Ortholog-Groups

To identify the names of genes included in each ortholog-group, predictions from two sources were incorporated, namely, the genome annotation file and names predicted by PANNZER2 (Törönen et al., 2018). First, the name of each gene, if any, was extracted from the genome annotation files. For prediction by PANNZER2, the peptide sequence of the longest isoform of each gene was used, and the predicted gene names and GO terms were retrieved. Predicted GO terms were mapped to GOslim terms (go.obo release version 2020–03–23) using GOATOOLS (Klopfenstein et al., 2018). Development-related ortholog-groups in Figure 5C were defined as those including genes with the GOslim term of “anatomical structure development” (GO:0048856). For Hox ortholog-groups (Figure 5C, left), predictions from both sources were manually checked because not every single *Hox* gene was conserved in all species.

#### 4.7.2 Tendency of Temporal Pleiotropy

For each category of vertebrate DCOs, the proportion of ortholog-groups showing different degrees of temporal pleiotropy across species was calculated. Since the number of sampled developmental stages was not uniform in different species, for each ortholog-group, the percentage of developmental stages where it is expressed (defined as TPM ≥1) in each species was first calculated. The mean percentage across the six vertebrate species was then calculated for each ortholog-group, and the whole range of mean percentage values was binned into ten 10% ranges to be plotted. For each category, the ratio of ortholog-groups with a mean percentage value within each bin is shown in Figure 5E. In other words, ortholog-groups inside the 100% bin (blue) indicate that they are expressed in all the sampled developmental stages in all six vertebrate species. Similarly, those inside the 90% bin (light blue) are expressed in 90–99% of all the sampled developmental stages on average across species. Thus, ortholog-groups showing a higher degree of temporal pleiotropy are shown towards the right end, while those with a lower degree of pleiotropy are towards the left side.

### 4.8 Read-Depth Normalization Between Species

To normalize read depth between samples of different species, the maximum possible number of reads was selected while keeping the same reads-to-exome-size ratio in all species. Best-hits were first selected using samtools (version 1.12; single-end samples: samtools view -F 4 | samtools view -F 256; paired-end samples: samtools view -f 2 | samtools view -F 256) (Li et al., 2009). Random pick-up of reads was performed from the BED file containing all mapped best-hit reads. For paired-end samples, only one read was reported for the BED file using the bamtobed function with the “-bedpe” option from bedtools (version 2.30.0) (Quinlan and Hall, 2010). The ratio of all best-hit reads to exome size in each sample was calculated, and the smallest ratio was selected as the baseline (Lv_60cell sample) to calculate the number of reads needed for each species (see Supplementary Table S18 for the calculated results). We tried to calculate exome size using the original genome annotation file as well as a version with all the untranslated regions (UTRs) removed to minimize unintentional bias, since only some of the species were annotated with UTRs (Supplementary Figure S15). To remove UTRs from annotation files, the script “gff3_file_UTR_trimmer.pl” from PASApipeline (Haas et al., 2003) was used. To calculate exome size, the command line “awk '{if ($3 = = "exon"){print $0}}' $gffname | gff2bed - | bedops -m - | awk ‘BEGIN{FS = "\t"; count = 0}{count = count + ($3-$2)}END{print count}'” with the BEDOPS tool (version 2.4.39) (Neph et al., 2012) was used.

### 4.9 Software and Computation Environment

Bioinformatics analyses were performed using in-house R (4.0.3) (R-Core-Team, 2020), Python (3.7), and shell scripts, together with the software and packages summarized in Supplementary Table S17.

## Data Availability

The datasets presented in this study can be found in online repositories. The names of the repository/repositories and accession number(s) can be found below: https://www.ncbi.nlm.nih.gov/, PRJNA749373.

## Supplementary Material

Supplementary Table S19 695 DCOs (derivedness-correlative ortholog-groups) showing negative correlations across six vertebrate species.

Supplementary Table S20 230 negative DCOs across eight chordate species.

Supplementary Table S21 2,414 negative DCOs across three echinoderm species.

## Abbreviations

Aj, sea cucumber (*Apostichopus japonicus*); Cg, oyster (*Crassostrea gigas*); DCO, derivedness-correlative ortholog-groups; Mm, mouse (*Mus musculus*); Gg, chicken (*Gallus gallus*); Ps, Chinese soft-shelled turtle (*Pelodiscus sinensis*); Xl, African clawed frog (*Xenopus laevis*); Dr, zebrafish (*Danio rerio*); Ol, medaka (*Oryzias latipes*); Ci, Vase tunicate (*Ciona intestinalis*); Bf, amphioxus (*Branchiostoma floridae*); Oj, feather star (*Anneissia japonica*); Lv, green sea urchin (*Lytechinus variegatus*); Sp, purple sea urchin (*Strongylocentrotus purpuratus*).
