# Supplementary material for: Derivedness Index for Estimating Degree of Phenotypic Evolution of Embryos: A Study of Comparative Transcriptomic Analyses of Chordates and Echinoderms
Source: Front Cell Dev Biol. 2021 Nov 26;9:749963. doi: 10.3389/fcell.2021.749963 (PMC8661034; doi:10.3389/fcell.2021.749963)
Supplement: Supplementary file 3 [file DataSheet1.pdf]

Supplementary Material

1 Supplementary Figures and Tables

1.1 Supplementary Figures

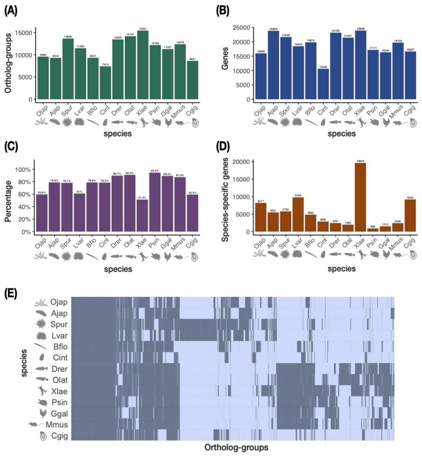

Supplementary Figure 1. Statistics of ortholog-group prediction by PorthoMCL. A total of 22,699 orthogroups were predicted for the 13 species. (A) Number of ortholog-groups with genes of each species. (B) We noticed that species-specific genes are not automatically included in the orthoMCL

or PorthoMCL default outputs. In other words, the default outputs are gene families with genes in at least two species. This plot shows the number of genes of each species included in the default prediction table. (C) Percentage of protein coding genes (in the genome) covered by ortholog-groups identified by porthomcl. (D) Number of non-paralogous species-specific genes of each species that were detected (TPM>0) in the developmental transcriptomic datasets. (E) A simplified visualization of orthogroup prediction results. Each dark pixel indicates that an ortholog-group includes genes (1 or more) in that species, whereas a light pixel indicates that an orthogroup does not have predicted genes in that species. Species abbreviation are as follows: *Ojap*: feather star; *Ajap*: sea cucumber; *Spur*: purple sea urchin; *Lvar*: green sea urchin; *Bflo*: amphioxus; *Cint*: tunicate; *Drer*: zebrafish; *Olat*: medaka; *Xlae*: frog; *Psin*: soft-shelled turtle; *Ggal*: chicken; *Mmus*: mouse; *Cgig*: oyster.

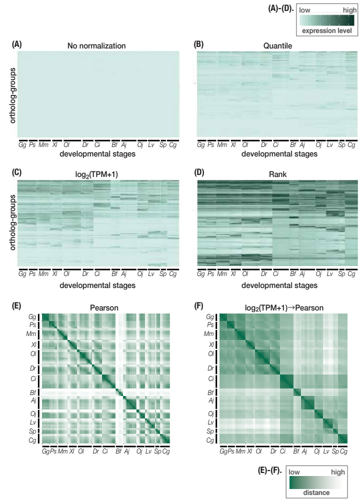

Supplementary Figure 2. Normalization of ortholog-group-based expression table. (A)-(D). Normalization of expression data. Expression data are aligned from early to late developmental stages in each species. These figures show that normalization of the expression data is necessary to increase its overall resolution for comparison between each other. Higher expression is represented by darker color in all four plots. (A) The original ortholog-group-based expression table without normalization. This overall plain image implies that the unprocessed expression table is dominated by a few extremely high values. (B) Quantile-normalized table; (C) log2(TPM+1)-transformed table; (D) ascending rank-transformed table (note: taking rank is the first step in calculating Spearman's

correlation coefficient). Resolution of the visualized expression table is higher if the data are normalized. Developmental stages of the same species show more similar ortholog-groups expression patterns after normalization. (E)-(F). Distance matrix before and after normalization. (E) Distance matrix calculated from 1-Pearson's correlation coefficient without normalization of expression data. (F) Distance matrix using the same distance calculation method (1-Pearson's correlation coefficient), but with a preprocessing step of log-normalizing the expression data. Similarity between developmental stages of the same species increased.

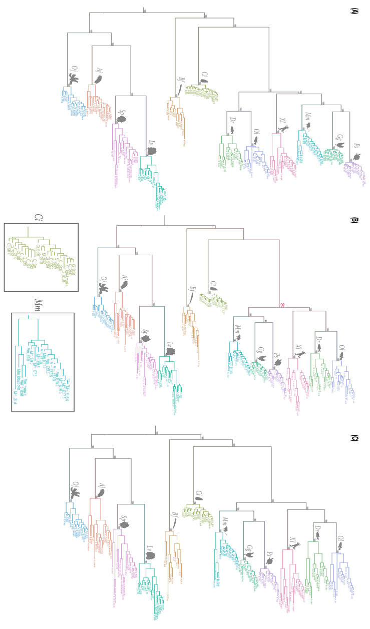

Supplementary Figure 3. Selected trees based on slightly-modified, alternative methods. (A) Expression of ortholog-groups calculated by sum-expression of paralogs. This tree is similar to that inferred from mean-expression of paralogs. All supported values marked are 100 (topology supported by 100 BRI, biological replicates-included, trees). (B) Tree inferred by Fitch-Margoliash criterion. The topology of this tree (((*Dr*,*Ol*),*Xl*),((*Gg*,*Ps*),*Mm*)) is not consistent with phylogeny inferred from genomic sequences. However, 100 BRI-trees support the topology ((*Dr*,*Ol*),(*Xl*,(*Mm*,(*Gg*,*Ps*)))), which is consistent with genomic sequence-based phylogeny. This discrepancy is marked by the red asterisk (*), but the underlying reason is unknown. In addition, the topology of the mouse (*Mm*) and the tunicate (*Ci*) clades are not the same as that shown in Main Figure 3A, with mouse E9.5 and tunicate stage 14 being the least derived stages. (C) Detected ortholog-groups are defined as TPM >= 1 (Expression less than this threshold is set to zero), but the tree topology became inconsistent with genomic sequences-based phylogeny.

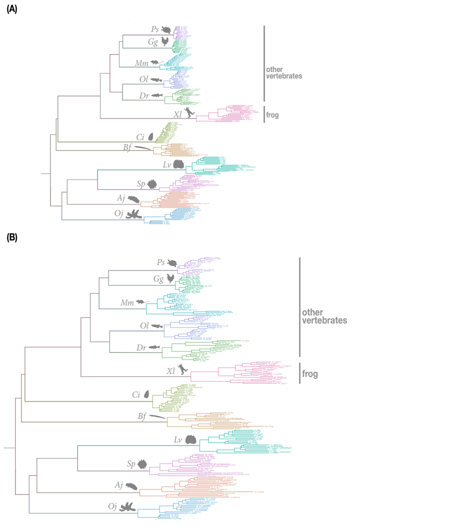

Supplementary Figure 4. Derivedness tree considering species-specific genes. (A) Expression levels of all genes were considered. (B) Genes with expression cutoff at TPM≥1 were considered. However, both trees violated criterion 2 (consistent with known phylogeny) as *X. laevis* became the outgroup of the other vertebrate species. Notably, among the 19,644 detected species-specific genes in *X. laevis*, 7,879 genes were lowly expressed (max TPM<1).

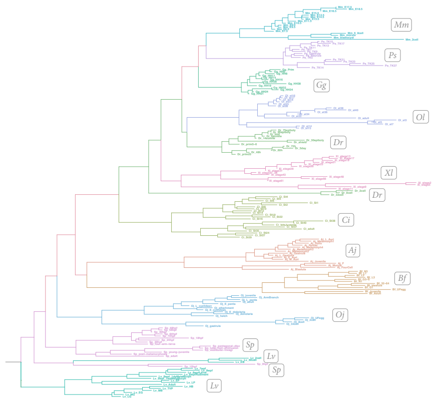

Supplementary Figure 5. Tree considering expression of only 1:1 orthologs. However, embryos of the same species (denoted by the same color) did not cluster together in sea urchins (*Lv*, *Sp*) and zebrafish (*Dr*). Species abbreviations are shown in squares.

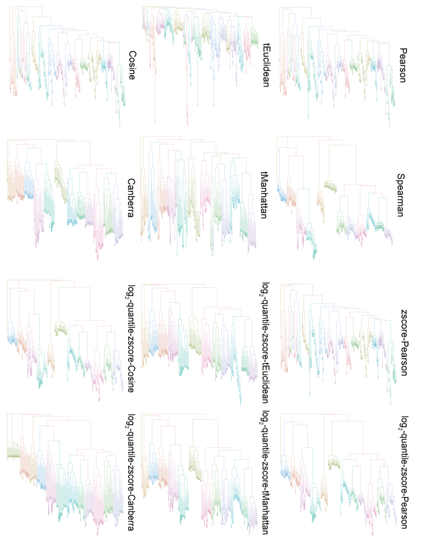

Supplementary Figure 6. Trees inferred from other distance methods. Embryos of the same species are denoted by the same color in each of the trees.

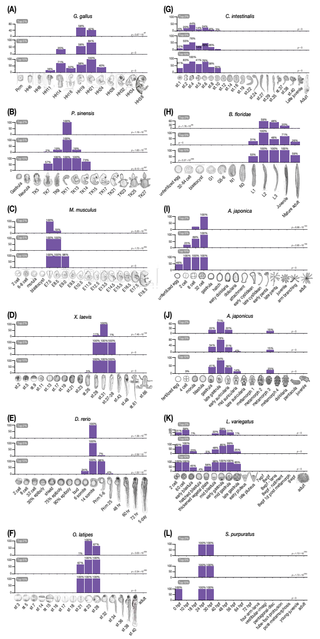

Supplementary Figure 7. Least derived stages identified as being within top 2% (top), 5% (middle), and 10% (bottom) lowest derivedness index in each species. The least derived developmental process may span multiple embryonic stages, which could be reflected in several embryos having similarly low derivedness indices. 100 random biological replicates-included (BRI) trees were utilized to get statistical support. For each BRI tree, the range of derivedness index of embryos of each species was first calculated, and stages within the lowest-2/5/10% range were marked. The percentage of the number of times each developmental stage was marked among the 100 BRI trees was then plotted for each species (Fisher's exact test). The result showed consistent tendency with that shown in Figure 4, with mid-embryonic, organogenesis stage in vertebrates and gastrula in echinoderms (except feather star) being the least derived.

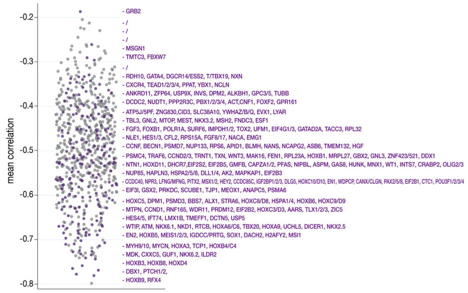

Supplementary Figure 8. DCOs (derivedness-correlative ortholog-groups) showing negative correlations across all six vertebrate species (total: 695) with predicted development-related functions (total: 201; points highlighted in purple). Y-axis: mean correlation value across the six vertebrate species. Each 0.1 range is further divided into five bins, and the predicted names of ortholog-groups within each bin are shown aside.

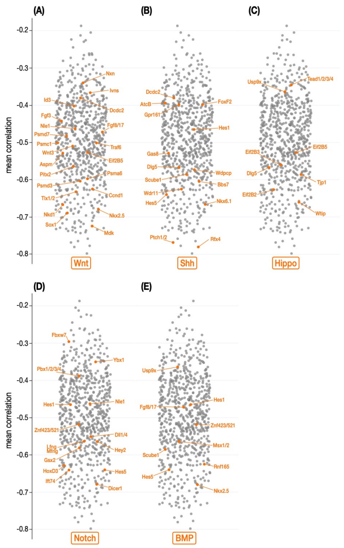

Supplementary Figure 9. Negative DCOs across vertebrate species with predicted function involved in signal transduction of (A) Wnt, (B) Shh, (C) Hippo, (D) Notch, and (E) BMP. Locations of points

appear to differ due to the jittering function when plotting; the horizontal locations (representing the mean correlation value) remain the same for the same point in all plots.

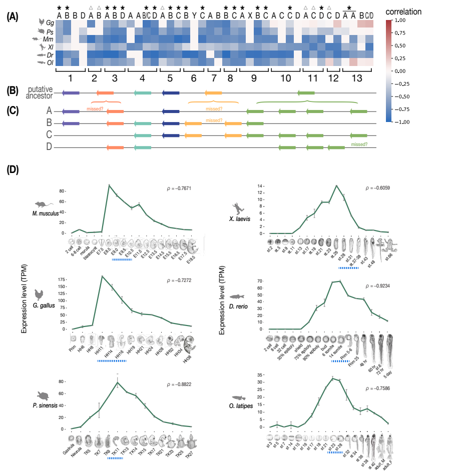

Supplementary Figure 10. Derivedness index-expression correlation analysis of Hox ortholog-groups in vertebrates. (A) Visualization of correlation coefficient of each Hox ortholog-groups in each vertebrate species (blue: negative correlation; red: positive correlation). The expression of most Hox ortholog-groups, especially anterior and mid Hox ortholog-groups (Hox1-9), showed strong negative correlation with derivedness index, suggesting that Hox genes could be involved in characterizing the least derived stages in vertebrate embryogenesis (*: negative correlation in all six species; △: negative correlation in five out of the six species). (B) Representation of Hox genes in the putative bilaterian ancestor (Prince et al., 1998). Arrow direction indicates 5' to 3'. (C) Hox genes conserved in the 6 vertebrate species. Those marked with "missed" were either possibly missed by the genome assembly in one species or not detected as expressed in the transcriptomic dataset. For

example, HoxA2 was missed in the genome assembly of *P. sinensis* (turtle). HoxA7 was not found in the genome assembly of *D. rerio* (zebrafish) while HoxB7, *O. latipes* (medaka). HoxD13 was missed in *O. latipes* (medaka). HoxA13 was separated into two ortholog-groups, one grouped with the fish-specific paralog *Dr*- and *Ol*-HoxA13A and the other one with *Dr*- and *Ol*-HoxA13B. (D) Expression of HoxB9 (the Hox ortholog-groups showing the strongest negative correlation with derivedness index) along development in vertebrate species. Its expression peaks around the least derived stage in all six species. (Least derived stages marked with dashed underlines; error bars represent s.d. of expression among biological replicates; ρ, Spearman's correlation coefficient.)

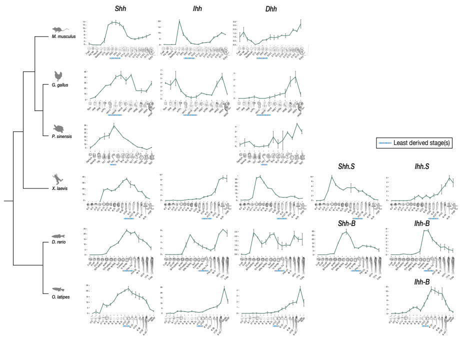

Supplementary Figure 11. Expression of *hedgehog* genes in vertebrates. The hedgehog family includes three genes (*Shh*, Sonic hedgehog; *Ihh*, Indian hedgehog; *Dhh*, Desert hedgehog), and was ambiguously classified into two ortholog-groups (1139 and 10783). We manually checked the gene name of individual genes from the genome annotation files and grouped them accordingly. *Shh* showed strong negative correlation with derivedness index (with high expression around the least derived stages in each species). Error bars represent s.d. of expression among biological replicates.

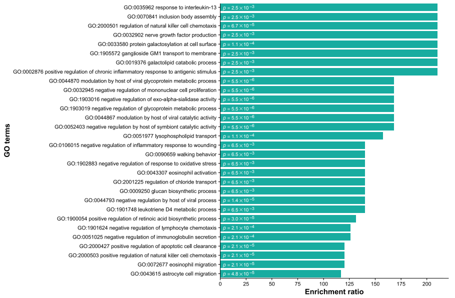

Supplementary Figure 12. Gene Ontology enrichment analysis of vertebrates-conserved positive DCOs. These ortholog-groups are more involved in immune and metabolic functions. Shown here are top 30 GO categories with the highest enrichment ratio and corrected $p < 0.05$ (p-value shown on each corresponding bar; false discovery rate ≤ 0.05 with Benjamini-Hochberg correction for multiple comparisons). Gene Ontology enrichment analysis was performed using GOATOOLS (Klopfenstein et al., 2018) with GO terms predicted by PANNZER2 (Törönen et al., 2018).

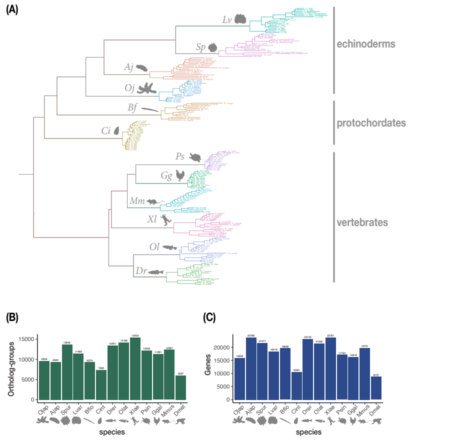

Supplementary Figure 13. Derivedness tree with *Drosophila* as the outgroup. (A) In this tree, the amphioxus (*Bf*) and the tunicate (*Ci*) cluster with the echinoderm species, which violates criterion 2 (consistent with known phylogeny). (B-C). Statistics of ortholog-group prediction by PorthoMCL with *Drosophila* (abbreviation: *Dmel*) as the outgroup. (B) Number of ortholog-groups with genes of each species. (C) Number of genes of each species included in the ortholog-group prediction. In contrast to using oyster as the outgroup (Supplementary Figure 1), considerably fewer genes in the *Drosophila* genome (8,727; oyster: 16,627) could be identified as orthologous genes of deuterostome genes.

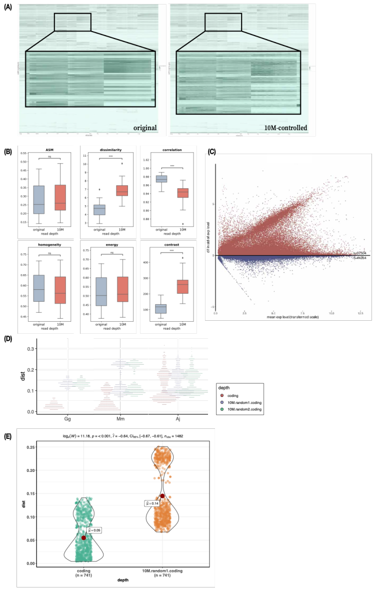

Supplementary Figure 14. Analysis of 10M depth-controlled expression data. Read depth tends to affect distance calculation between transcriptomes. (A) Visualization of ortholog-group-based expression table. While the original (without read-depth control, left) expression table showed high continuity along development, the 10M depth-controlled expression table (right) seemed to have incorporated more "noises" in the image, indicating that expression may largely differ when read-depth control is implemented. The orders of ortholog-groups (along the vertical axis) are the same in the two images while the order was determined by a clustering algorithm performed on the original table. (B) Smoothness analysis of the two images. Three out of the six descriptors showed that the original table was significantly smoother than the 10M depth-controlled expression table (two-sample t-test) (blue: original; red: 10M depth-controlled). (C) Difference in standard deviation of expression among biological replicates. More ortholog-groups in the 10M depth-controlled table (red) showed higher standard deviation in expression among biological replicates than that in the original table (blue). Each point represents one ortholog-group in one developmental stage. Y-axis: standard deviation of each ortholog-group in the 10M depth-controlled table subtract that in the original table. (D-E) Distribution of pairwise distances between embryonic transcriptomes within each species (*Gg*: chicken; *Mm*: mouse; *Aj*: sea cucumber). In (D), Two random replicates of 10M depth-controlled (blue and green) were analyzed and showed similar tendencies. Distances calculated from the 10M depth-controlled data tended to be larger than those calculated from the original expression data (red). In (E), only the distances of the mouse are shown, with statistical analysis ($\hat{\mu}$, distribution mean; non-parametric Mann–Whitney–Wilcoxon test). Taken together, 10M-depth control tends to show significant increase in distances and derivedness index (Main Figure 6A).

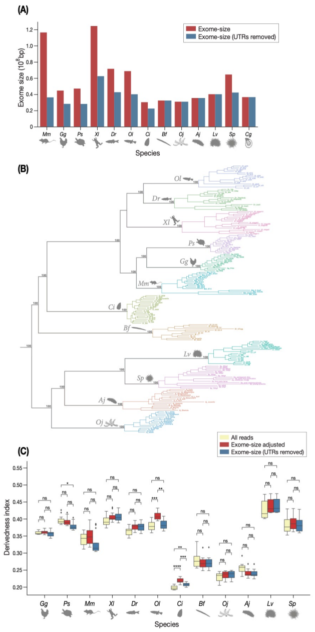

Supplementary Figure 15. Derivedness tree with exome size-adjusted read depth (i.e., certain million reads per kb exon for all species). (A) Exome size of each species. Only some of the species were annotated with UTRs. To avoid this genome annotation bias, exome sizes when UTRs are removed are shown in blue (see also Supplementary Table 18). (B) Tree based on expression data with exome sizes-adjusted depth. However, this tree violates criterion 2 (consistent with known phylogeny) as the frog (*Xl*) clusters with the fish species (*Dr*, *Ol*). (C) The range of derivedness indices for embryos of each species. Only few species are affected after the exome-size read depth control or when UTRs are removed from the genome annotation files. (Mann–Whitney–Wilcoxon test)

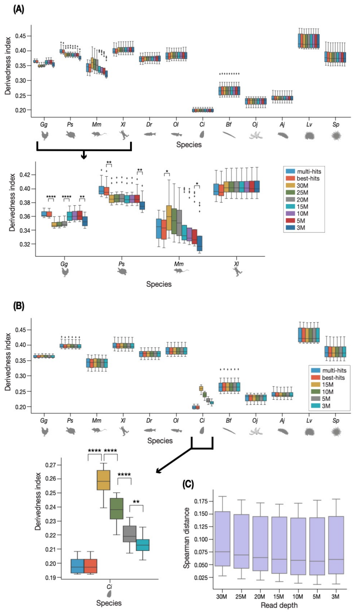

Supplementary Figure 16. Transcriptomic derivedness index could potentially be influenced by RNA-seq read depth. (A) To study the effect of read depth, a down-sampling of read depth in the mouse (*Mm*) dataset from 30M through 25M, 20M, 15M, 10M, 5M, and 3M was performed (while keeping the depth of the datasets of all other species unchanged to compare with Figure 3A). Reads were randomly picked from best-hit mapped reads (the total number of all best-hit mapped reads was around 30M for most of the mouse datasets). The result showed that derivedness index tends to decrease when read depth decreases, and those of phylogenetically related species, such as chicken (*Gg*) and turtle (*Ps*), were also affected. (B) Similar effect was observed when down-sampling reads in the tunicate (*Ci*) dataset (from 15M to 10M, 5M, and 3M). However, even when down-sampling was done to 3M reads (the teal box), the range of derivedness indices was still significantly greater than that when all best-hit reads were retained (the orange box). (Mann-Whitney U test, $p < 0.01$) These results are consistent with those shown in Figure 6A and Supplementary Figure 14. (C) Pairwise distances (1-Spearman's correlation correlation) among mouse samples only slightly differed when down-sampling was performed (Kruskal-Wallis test, $p < 0.01$).

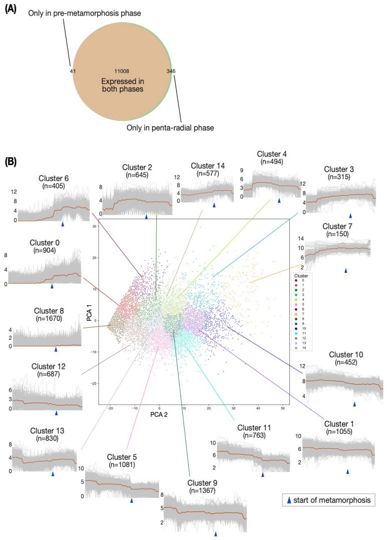

Supplementary Figure 17. Pre-metamorphosis and the penta-radial phase deploy similar sets of genes but at different expression levels in *L. variegatus*. (A) The majority of ortholog-groups (11,008) are expressed in both the pre-metamorphic and the penta-radial phases. Only a few ortholog-groups are specific to either phase (41 and 346, respectively). (B) K-means (k=15) analysis of expression levels from early to late developmental stages of ortholog-groups supports that most ortholog-groups are

expressed at different levels in pre-metamorphic and penta-radial phases. Each cluster represents ortholog-groups that tend to show similar expression dynamics from early to late development. For instance, ortholog-groups in cluster 8 are mostly lowly expressed in both developmental phases while those in cluster 6 tend to be expressed more highly in the penta-radial phase. [x-axis: early-to-late development, with the appearance of penta-radial structures marked by the blue triangle (7wpf); y-axis: expression level $\log_2$(TPM+1); Red line: median expression level of ortholog-groups of the cluster.] These results tend to support that the differences in transcriptomic derivedness indices of the pre-metamorphic and the penta-radial developmental stages could be partly attributed to ortholog-groups expressing at different levels rather than deploying different sets of genes during the two phases. K-means and PCA analyses were performed using scikit-learn in Python.

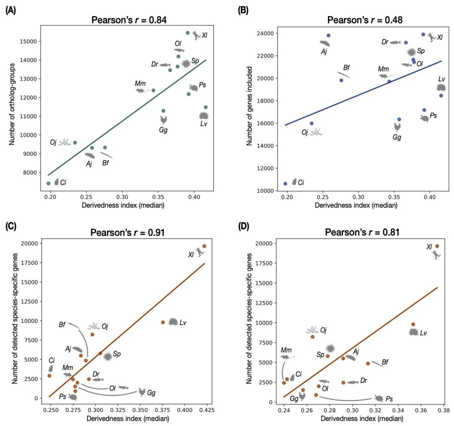

Supplementary Figure 18. Correlation tests between derivedness index of each species and ortholog-group statistics (those shown in Supplementary Figure 1). The median of the derivedness indices of all developmental stages of each species was utilized to represent the species. (A) Number of ortholog-groups (Supplementary Figure 1A) shows a moderately strong correlation with derivedness index. (B) Number of genes (Supplementary Figure 1B; excluding species-specific genes) shows a weak correlation with derivedness index. (C-D) When species-specific genes are considered in tree inference (Supplementary Figure 4), the number of detected species-specific genes (C: no expression level cutoff; D: expression cutoff at TPM≥1) is strongly correlated with the measured derivedness index. ($p < 0.01$ for all panels)

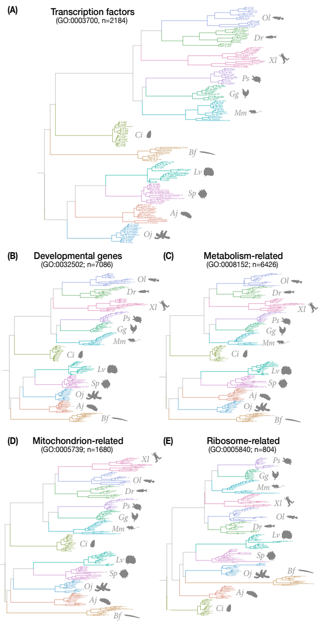

Supplementary Figure 19. Tree inferred with different categories of ortholog-groups: (A) transcription factors; (B) developmental genes; (C) metabolism-related genes; (D) mitochondrion-related genes; (E) ribosome-related genes. All of these trees exhibited a samples-clustered-by-species topology. Among these, the tree with transcription factors is the one that most resembles the tree inferred with all ortholog-groups. In this tree, tunicate embryos showed the least derived indices, which was consistent with the tree using all ortholog-groups, and the topology only deviated slightly from the known phylogeny (the frog clustered with the fish species). Notably, the green sea urchin (*Lv*) and the purple sea urchin (*Sp*) showed similar branch lengths in the tree with transcription factors whereas larger differences between them were observed in all the other trees. This suggests that the differences in overall derivedness of the two sea urchins could be attributed to genes other than transcription factors.

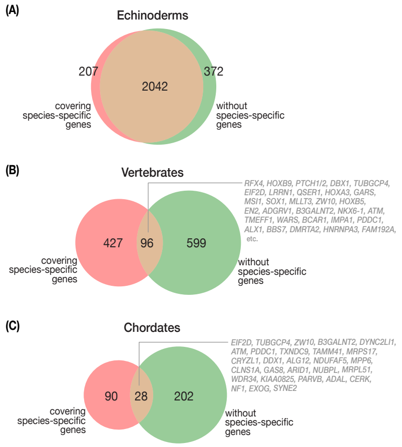

Supplementary Figure 20. DCO (Derivedness-correlative ortholog-groups) analysis using the tree covering species-specific genes (Supplementary Figure 4; expression threshold having a negligible effect). Compared with the results from the tree excluding species-specific genes (those shown in Main Figures 3-5), the extracted DCOs with negative correlation across (A) all 3 echinoderm species showed a high degree of overlap, which is consistent with the observation that derivedness index of each developmental stage in echinoderm species did not change drastically in the tree covering species-species genes. However, larger differences were observed in (B) vertebrates and (C) chordates. This could be due to the differences in derivedness indices of developmental stages of mouse and zebrafish between the two trees. Extracted ortholog-groups that could be extracted from both trees in vertebrates and chordates are highlighted (ordered by negative correlation coefficients).

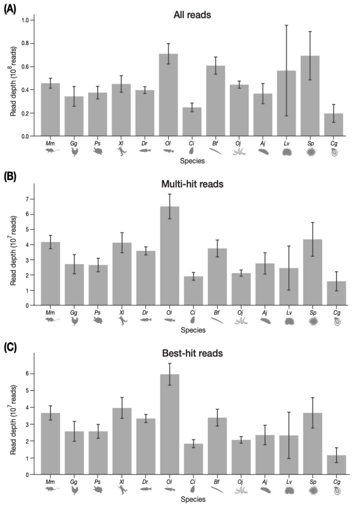

Supplementary Figure 21. Read depth of samples. (A) Number of all raw reads; (B) Number of reads that could be mapped to the respective genomes, including multi-hit reads (selected by "samtools view -F 4" or "samtools view -f 2" for single-end or paired-end samples, respectively); (C) Number of best-hit reads (further selected by "samtools view -F 256" from multi-hit BAM files). Error bars represent standard deviations of read depths for samples of each species.

1.2 Supplementary Tables

Supplementary Table 1. Developmental stages included in the study: feather star (*Anneissia japonica*). To avoid confusion with the sea cucumber, which has a very similar scientific name, the species abbreviation for feather star, *Oj*, was taken from its previous scientific name, *Oxycomanthus japonicus* (Müller, 1841).

Species abbre-viation	General name	Developmental stages	Stage abbreviation	Source
Oj	Feather star	Unfertilized egg	UFegg	(Li et al., 2020)
		2 cells (1.5 h post fertilization, hpf)	2cell	
		8 cells (2.5 hpf)	8cell	
		32 cells (3.5 hpf)	32cell	
		Gastrula (8 hpf)	gastrula	
		Hatching stage (17 hpf)	hatch	
		Early doliolaria (24 hpf)	early_doliolaria	
		Mid-late doliolaria (36 hpf)	doliolaria	
		Attachment stage (3-4 days pf)	attachment	
		Early cystidean (4-7 days pf)	early_cystidean	
		Late cystidean (7-9 days pf)	late_cystidean	
		Early pentacrinoid (3 weeks pf)	early_penta	
		Late pentacrinoid (1.5 months pf)	late_penta	
		Juvenile (2.5 months pf)	juvenile	
		Arm branching stage (6-7 months pf)	armBranch	
		Adult (9 months pf)	adult	

Supplementary Table 2. Developmental stages included in the study: sea cucumber (*Apostichopus japonicus*).

Species abbre-viation	General name	Developmental stages	Stage abbreviation	Source
Aj	Sea cucumber	Fertilized egg	Fertilized_egg	(Li et al., 2018)
		4 cells (2 hpf)	FourCell	
		Morula (6 hpf)	Morula	
		Blastula (14 hpf)	Blastula	
		Gastrula (29 hpf)	Gastrula	
		Late gastrula (34 hpf)	L_Gastrula	
		Early auricularia larva (48 hpf)	E_Auri	
		Mid-auricularia larva (69 hpf)	M_Auri	
		Late auricularia larva (15 days post fertilization, dpf)	L_Auri	
		Metamorphosis 1 ~ 4 (17-19 dpf)	Metamorph1 Metamorph2 Metamorph3 Metamorph4	
		Doliolaria larva (19 dpf)	Dolio	
		Pentactula larva (27 dpf)	Pentac	
		Juvenile (51 dpf)	Juvenile	

Supplementary Table 3. Developmental stages included in the study: green sea urchin (*Lytechinus variegatus*).

Species abbre-viation	General name	Developmental stages	Stage abbreviation	Source
Lv	Green sea urchin	2 cells (1 hpf)	2cell	(Li et al., 2020)
		60 cells (2.5 hpf)	60cell	
		Early blastula (4 hpf)	EB	
		Hatched blastula (7 hpf)	HB	
		Thickened vegetal plate (10 hpf)	TVP	
		Mesenchyme blastula (12 hpf)	MB	
		Early gastrula (13 hpf)	EG	
		Mid gastrula (15 hpf)	MG	
		Late gastrula (18 hpf)	LG	
		Early pluteus (36 hpf)	EP	
		Late pluteus (48 hpf)	LP	
		7 weeks post fertilization (7 wpf)	7wpf	
		8 weeks post fertilization (8 wpf)	8wpf	
		8 wpf, non-rudiment part	8wpfLarva	
		8 wpf, rudiment part	8wpfRudiment	
		1 day post metamorphosis	8wpf_1dpMetaMorph	
		1 week post-metamorphosis	9wpf_1wpMetaMorph	
		Adult	Adult	

Supplementary Table 4. Developmental stages included in the study: purple sea urchin (*Strongylocentrotus purpuratus*).

Species abbre-viation	General name	Developmental stages	Stage abbreviation	Source
Sp	Purple sea urchin	Unfertilized egg (0 hpf)	0hpf	(Tu et al., 2012, 2014)
		Cleavage (10 hpf)	10 hpf	
		Hatched blastula (18 hpf)	18hpf	
		Mesenchyme blastula (24 hpf)	24hpf	
		Early gastrula (30 hpf)	30hpf	
		Mid gastrula (40 hpf)	40hpf	
		Late gastrula (48 hpf)	48hpf	
		Prism (56 hpf)	56hpf	
		Late prism (64 hpf)	64hpf	
		Pluteus (72 hpf)	72hpf	
		Four-arm larval stage	four-arm-larva	
		Vestibular invagination stage	vestibular-invagi	
		Pentagonal disc stage	pentagonal-disc	
		Tube-foot protrusion stage	tube-foot-protrusion	
		Post-metamorphosis	post-metamorphosis	
		Young juvenile	young-juvenile	
		Adult	adult	

Supplementary Table 5. Developmental stages included in the study: amphioxus (*Branchiostoma floridae*). Staging was performed as described in (Hirakow and Kajita, 1990, 1991, 1994; Yu and Holland, 2009).

Species abbre-viation	General name	Developmental stages	Stage abbreviation	Source
Bf	Amphio-xus	Unfertilized egg	UFegg	(Hu et al., 2017)
		32-64 cells	32-64	
		Blastula	blastula	
		Early gastrula	G1	
		Late gastrula	G5-6	
		Early neurula	N1	
		Later neurula	N3	
		Early knife-shaped larva	L1	
		Open mouth larva	L2	
		Two gill slit larva	L3	
		0.5-1cm-long animal	Juvenile	
		Adult with mature oocytes	MatureFemale	
		Adult with mature spermatocytes	MatureMale	

Supplementary Table 6. Developmental stages included in the study: ascidian tunicate (*Ciona intestinalis*). Staging was performed as described in (Chiba et al., 2004; Hotta et al., 2007).

Species abbre-viation	General name	Developmental stages	Stage abbreviation	Source
Ci	Ascidian tunicate	Fertilized egg (1 cell)	St1	(Hu et al., 2017)
		2 cells	St2	
		8 cells	St4	
		16 cells	St5	
		32 cells	St6	
		64 cells	St8	
		Initial gastrula	St10	
		Mid gastrula	St12	
		Early neurula	St14	
		Late neurula	St16	
		Early tailbud	St19	
		Mid tailbud	St22	
		Late tailbud	St24	
		Early swimming larva	St27	
		Late swimming larva	St29	
		Early rotation	St35	
		Late rotation	St37	
		Early 1st ascidian (Early juvenile I)	St38	
		Late 1st ascidian (Mid juvenile I)	St40	
		2nd ascidian (Late juvenile)	lateJuvenile	
		Adult	adult	

Supplementary Table 7. Developmental stages included in the study: zebrafish (*Danio rerio*). Staging was performed as described in (Kimmel et al., 1995).

Species abbre-viation	General name	Developmental stages	Stage abbreviation	Source
Dr	Zebra-fish	2 cells	2cell	(Hu et al., 2017)
		8 cells	8cell	
		32 cells	32cell	
		30% epiboly	30epiboly	
		Shield stage (gastrula)	shield	
		75% epiboly (gastrula)	75epiboly	
		90% epiboly (gastrula)	90epiboly	
		Bud stage (gastrula)	bud	
		6-somite (segmentation)	6somite	
		14-somite (segmentation)	14somite	
		Prim5-6 (pharyngula)	prim5-6	
		Prim25 (pharyngula)	prim25	
		Long-pec	48h	
		Pec-fin	60h	
		Protruding-mouth	72h	
		5 day	5day	

Supplementary Table 8. Developmental stages included in the study: medaka (*Oryzias latipes*). Staging was performed as described in (Kinoshita et al., 2012).

Species abbre-viation	General name	Developmental stages	Stage abbreviation	Source
Ol	Medaka	2 cells (1 h 5 min)	st3	(Ichikawa et al., 2017)
		8 cells (2 h 20 min)	st5	
		32 cells (3 h 30 min)	st7	
		Pre-mid gastrula stage (15 h)	st14	
		Mid gastrula stage (17 h 30 min)	st15	
		Late gastrula stage (21 h)	st16	
		Early neurula stage (1 d 1 h)	st17	
		Late neurula stage (1 d 2 h)	st18	
		6 somite stage (1 d 10 h)	st21	
		12 somite stage (1 d 17 h)	st23	
		30 somite stage (2 d 16 h)	st28	
		Somite completion stage (4 d 5 h)	st32	
		Pectoral fin blood circulation stage (5 d 1 h)	st34	
		Heart development stage (6 d)	st36	
		Spleen development stage (8 d)	st38	
		1st fry stage	st40	
		Adult (male)	adultM	
		Adult (female)	adultF	

Supplementary Table 9. Developmental stages included in the study: African clawed frog (*Xenopus laevis*). Staging was performed as described in (Nieuwkoop and Faber, 1994).

Species abbre-viation	General name	Developmental stages	Stage abbreviation	Source
Xl	African clawed frog	2 cells	stage2	(Hu et al., 2017)
		16 cells	stage5	
		Blastula	stage9	
		Early gastrula	stage11	
		Small yolk plug stage	stage13	
		Late neural fold	stage17	
		4-6 somites	stage19	
		Neural tube closure	stage21	
		12 somites	stage23	
		Stage 26	stage26	
		20-22 somites	stage28	
		Tail bud	stage31	
		Stage 37-38	stage37_38	
		Visible lateral line system	stage43	
		Forelimb bud	stage48	
		Tentacle shortened	stage61	
		Very small triangle tail	stage66	

Supplementary Table 10. Developmental stages included in the study: soft-shelled turtle (*Pelodiscus sinensis*). Staging was performed as described in (Tokita and Kuratani, 2001).

Species abbre-viation	General name	Developmental stages	Stage abbreviation	Source
Ps	Softshell turtle	Gastrula	Gastrula	(Wang et al., 2013; Hu et al., 2017)
		Neurula	Neurula	
		3-4 somites	TK5	
		7 somites	TK7	
		14 somites	TK9	
		27 somites	TK11	
		Long limb buds	TK13	
		TK14	TK14	
		Carapacial ridge	TK15	
		Distinct iris	TK17	
		Carapace pigmentation	TK21	
		TK23	TK23	
		Brownish body color	TK25	
		TK27	TK27	

Supplementary Table 11. Developmental stages included in the study: chicken (*Gallus gallus*). Staging was performed as described in (Hamburger and Hamilton, 1951).

Species abbre-viation	General name	Developmental stages	Stage abbreviation	Source
Gg	Chicken	Primitive streak	Prim	(Wang et al., 2013; Hu et al., 2017)
		HH6 (head fold)	HH6	
		HH8 (4 somites)	HH8	
		HH11 (13 somites)	HH11	
		HH14 (22 somites)	HH14	
		HH16 (26-28 somites)	HH16	
		HH19	HH19	
		HH21	HH21	
		HH24 (Toe plate)	HH24	
		HH28 (3 digits, 4 toes)	HH28	
		HH32	HH32	
		HH34 (Nictitating membrane)	HH34	
		HH38	HH38	

Supplementary Table 12. Developmental stages included in the study: mouse (*Mus musculus*). Staging was performed as described in (Kaufman, 1992).

Species abbre-viation	General name	Developmental stages	Stage abbreviation	Source
Mm	Mouse	2 cells	2cell	(Hu et al., 2017)
		6-8 cells	6_8cell	
		Morula	morula	
		Blastocyst	blastocyst	
		E7.5 (Neural plate)	E7.5	
		E8.5 (Turning)	E8.5	
		E9.0	E9.0	
		E9.5 (Forelimb bud)	E9.5	
		E10.5 (35-39 somites)	E10.5	
		E11.5 (Lens vesicle separated)	E11.5	
		E12.5	E12.5	
		E13.5	E13.5	
		E14.5 (56-60 somites)	E14.5	
		E15.5	E15.5	
		E16.5	E16.5	
		E17.5	E17.5	
		E18.5 (Long whiskers)	E18.5	

Supplementary Table 13. Developmental stages included in the study: oyster (*Crassostrea gigas*).

Species abbre-viation	General name	Developmental stages	Stage abbreviation	Source
Cg	Oyster	Eggs	E	(Zhang et al., 2012)
		2 cells (1 h 20 min)	TC	
		4 cells (1 h 32 min)	FC	
		Early morula (2 h 25 min)	EM	
		Morula (3 h 30 min)	M	
		Blastula (4 h 35 min)	B	
		Rotary movement (5 h 30 min)	RM	
		Free swimming (6 h 35 min)	FS	
		Early gastrula (7 h 35 min)	EG	
		Gastrula (8 h 30 min)	G	
		Trochophore (9 h 30 min – 14 h 35 min)	T1,T2,T3,T4,T5	
		Early D-shape larva (15 h 30 min – 16 h 35 min)	ED1,ED2	
		D-shape larva (17 h 35 min – 3.77 d)	D1,D2,D3,D4, D5,D6,D7	
		Early umbo larva (4.77 d – 6.75 d)	EU1,EU2	
		Umbo larva (7.75 d – 13.75 d)	U1,U2,U3, U4,U5,U6	
		Late umbo larva (14.73 d – 15.73 d)	LU1, LU2	
		Pediveliger (18.03 d – 18.19 d)	P1,P2	
		Spat (22.15 d)	S	
		Juvenile (215 d)	J	

Supplementary Table 14. Information of RNA-seq samples utilized for this study.

Species abbre-viation	General name	Accession number	Library preparation methods	Single-end (SE) / paired-end (PE)	Sequencing platform
Oj	Feather star	PRJNA553591	TruSeq	PE, 150 bp	Illumina Hiseq 4000
Aj	Sea cucumber	PRJNA553613	Quartz-Seq	SE, 100 bp	Illumina Hiseq 4000
Lv	Green sea urchin	PRJNA554218	TruSeq	PE, 100 bp	Illumina Hiseq 4000
Sp	Purple sea urchin	PRJNA81157	TruSeq-like (Mortazavi et al., 2008; Trapnell et al., 2010) with modifications	PE, 76 bp	llumina Genome Analyzer IIx
Bf	Amphioxus	DRA003460	TruSeq	SE, 100 bp	Illumina Hiseq 2000
Ci	Tunicate	DRA003460	TruSeq	SE, 100 bp	Illumina Hiseq 2000
Dr	Zebrafish	DRA003460	TruSeq	SE, 100 bp	Illumina Hiseq 2000
Ol	Medaka	DRA005309	TruSeq	PE, 100 bp	Illumina Hiseq 4000
Xl	Frog	DRA003460	TruSeq	SE, 100 bp	Illumina Hiseq 2000
Ps	Soft-shell turtle	DRA003460	TruSeq	SE, 100 bp	Illumina Hiseq 2000
Gg	Chicken	DRA003460	TruSeq	SE, 100 bp	Illumina Hiseq 2000
Mm	Mouse	DRA003460	Quartz-Seq (2-cell to blastocyst)	SE, 100 bp	Illumina Hiseq 2000
			TruSeq (E7.5 to E18.5)	SE, 100 bp	Illumina Hiseq 2000
Cg	Oyster	GSE31012	TruSeq-like (Zhang et al., 2012)	SE, 49 bp	Illumina Hiseq 2000

Supplementary Table 15. Genomes were utilized for RNA-seq mapping and ortholog-group prediction.

Species abbreviation	General name	Genome version	Source
Oj	Feather star	PRJNA553656	NCBI
Aj	Sea cucumber	ASM275485v1	NCBI
Lv	Green sea urchin	PRJNA553643	NCBI
Sp	Purple sea urchin	GCF_000002235.4	NCBI
Bf	Amphioxus	v18h27.r3_ref	LanceletDB
Ci	Tunicate	GCA_000224145.1	Ensembl
Dr	Zebrafish	GRCz10	Ensembl
Ol	Medaka	ASM223467v1	Ensembl
Xl	Frog	Xenla9.1_v1.8.3.2	Xenbase
Ps	Softshell turtle	GCA_000230535.1	Ensembl
Gg	Chicken	Gallus_gallus-5.0	Ensembl
Mm	Mouse	GRCm38	Ensembl
Cg	Oyster	oyster.v9	GigaDB

Supplementary Table 16. Descriptors of smoothness analysis (Gonzalez and Woods, 2007).

Descriptor	Formula	Range of values
Homogeneity	$\sum_{i=1}^{N}\sum_{j=1}^{N}\frac{p_{ij}}{1+\lvert i-j\rvert}$	[0, 1];<br>smoothest = 1
Dissimilarity	$\sum_{i=1}^{N}\sum_{j=1}^{N}p_{ij}\lvert i-j\rvert$	$[0, N-1]$;<br>smoothest = 0
Contrast	$\sum_{i=1}^{N}\sum_{j=1}^{N}p_{ij}(i-j)^2$	$[0, (N-1)^2]$;<br>smoothest = 0
Uniformity (Energy)	$\sum_{i=1}^{N}\sum_{j=1}^{N}p_{ij}^2$	[0, 1];<br>smoothest = 1
Correlation	$\sum_{i=1}^{N}\sum_{j=1}^{N}p_{ij}\left[\frac{(i-\mu_i)(j-\mu_j)}{\sigma_i\sigma_j}\right]$	$[-1, 1]$;<br>smoothest = 1<br>(perfect positive correlation between neighboring pixels)

Supplementary Table 17. Bioinformatics tools used in the study.

Analysis	Tools
RNA-seq mapping	sra-tools, trimmomatic (Bolger et al., 2014), FastQC,<br>samtools (Li et al., 2009), HISAT2 (v2.1.0) (Kim et al., 2019),<br>StringTie (v1.3.4d) (Pertea et al., 2015),<br>bedtools (Quinlan and Hall, 2010)
Ortholog-group prediction	PorthoMCL, orthoMCL (Li et al., 2003; Tabari and Su, 2017)
Expression data normalization	[R] base, Bioconductor (Huber et al., 2015),<br>preprocessCore (Bolstad, 2019)
Distance calculation	[Python] statistics, scipy.stats,<br>scipy.spatial.distance (Virtanen et al., 2020),<br>multiprocess (McKerns et al., 2012)<br>[R] stats
Distance matrix visualization	[R] ggplot2 (Wickham, 2016), cowplot, RColorBrewer, scales
Tree inference	[R] ape – nj, BIONJ, (Saitou and Nei, 1987; Gascuel, 1997; Paradis and Schliep, 2018),<br>FastME.bal, FastME.ols (Lefort et al., 2015),<br>Rphylip – Rfitch (Fitch-Margoliash)<br>(Fitch and Margoliash, 1967; Revell and Chamberlain, 2014),<br>phytools (Revell, 2011)<br>[Python] Bio.Phylo.Consensus (Talevich et al., 2012)
Tree visualization	[R] ggtree (Yu et al., 2016)
Smoothness analysis	[Python] skimage.io, skimage.feature.greycomatrix,<br>skimage.feature.greycoprops (Walt et al., 2014)
Statistical test	[R] base, ggstatsplot (Patil, 2021)<br>[Python] scipy.stats, statannot
Genomic analysis	PASA (Docker version) (Haas et al., 2003), BCBio,<br>bedops (Neph et al., 2012),<br>PANNZER2 (Törönen et al., 2018),<br>Bio.SeqIO, Bio.Seq, Bio.SeqRecord (Cock et al., 2009),<br>GOATOOLS (Klopfenstein et al., 2018)
Plotting	[Python] matplotlib (Hunter, 2007), seaborn (Waskom, 2021),<br>matplotlib-venn, plotly (Plotly, 2015), colorcet

Supplementary Table 18. Read depth was adjusted proportionally to the exome size of each species. The total exome size was calculated using the command line reported in the Methods section. As regions annotated with exons occasionally overlap with UTRs, the total exome size could change after the UTRs were removed from the genome annotation file (non-shaded columns). The number of reads for each species was calculated to maintain the same depth-to-exome size ratio for each species.

Species	Exome size in the original annotation file (bp)	Depth-controlled number of reads (Original)	Exome size in the annotation file with UTRs removed (bp)	Depth-controlled number of reads (UTRs removed)
Mm	116,671,536	15,795,970	36,499,719	4,941,638
Gg	44,887,066	6,077,187	28,473,497	3,854,981
Ps	47,344,196	6,409,854	28,388,868	3,843,523
Xl	124,613,914	16,871,276	62,658,081	8,483,176
Dr	71,753,612	9,714,605	42,868,042	5,803,835
Ol	68,896,411	9,327,774	40,334,262	5,460,790
Ci	30,517,304	4,131,688	22,685,000	3,071,285
Bf	32,595,114	4,413,000	32,595,114	4,413,000
Oj	31,151,795	4,217,591	31,151,795	4,217,591
Aj	35,666,769	4,828,866	35,666,769	4,828,866
Lv	40,383,687	5,467,482	40,383,687	5,467,482
Sp	64,735,250	8,764,401	42,457,298	5,748,225
Cg	36,798,484	4,982,087	36,798,484	4,982,087

Supplementary Table 19. 695 DCOs (derivedness-correlative ortholog-groups) showing negative correlations across six vertebrate species.

Supplementary Table 20. 230 negative DCOs across eight chordate species.

Supplementary Table 21. 2,414 negative DCOs across three echinoderm species.

2 Supplementary References

Bolger, A. M., Lohse, M., and Usadel, B. (2014). Trimmomatic: a flexible trimmer for Illumina sequence data. *Bioinformatics* 30, 2114–2120. doi:10.1093/bioinformatics/btu170.

Bolstad, B. (2019). *preprocessCore: a collectioin of pre-processing functions*. Available at: https://github.com/bmbolstad/preprocessCore.

Chiba, S., Sasaki, A., Nakayama, A., Takamura, K., and Satoh, N. (2004). Development of Ciona intestinalis juveniles (through 2nd ascidian stage). *Zool Sci* 21, 285–298. doi:10.2108/zsj.21.285.

Cock, P. J. A., Antao, T., Chang, J. T., Chapman, B. A., Cox, C. J., Dalke, A., et al. (2009). Biopython: freely available Python tools for computational molecular biology and bioinformatics. *Bioinformatics* 25, 1422–1423. doi:10.1093/bioinformatics/btp163.

Fitch, W. M., and Margoliash, E. (1967). Construction of phylogenetic trees. *Science* 155, 279–284. doi:10.1126/science.155.3760.279.

Gascuel, O. (1997). BIONJ: an improved version of the NJ algorithm based on a simple model of sequence data. *Mol Biol Evol* 14, 685–695. doi:10.1093/oxfordjournals.molbev.a025808.

Gonzalez, R. C., and Woods, R. E. (2007). “Regional descriptors: Texture,” in *Digital Image Processing* (Upper Saddle Rier, NJ: Pearson Prentice Hall), 827–839.

Haas, B. J., Delcher, A. L., Mount, S. M., Wortman, J. R., Jr, R. K. S., Hannick, L. I., et al. (2003). Improving the Arabidopsis genome annotation using maximal transcript alignment assemblies. *Nucleic Acids Res* 31, 5654–5666. doi:10.1093/nar/gkg770.

Hamburger, V., and Hamilton, H. L. (1951). A series of normal stages in the development of the chick embryo. *J Morphol* 88, 49–92. doi:10.1002/jmor.1050880104.

Hirakow, R., and Kajita, N. (1990). An electron microscopic study of the development of amphioxus, Branchiostoma belcheri tsingtauense: Cleavage. *J Morphol* 203, 331–344. doi:10.1002/jmor.1052030308.

Hirakow, R., and Kajita, N. (1991). Electron microscopic study of the development of amphioxus, Branchiostoma belcheri tsingtauense: The gastrula. *J Morphol* 207, 37–52. doi:10.1002/jmor.1052070106.

Hirakow, R., and Kajita, N. (1994). Electron microscopic study of the development of amphioxus, Branchiostoma belcheri tsingtauense: the neurula and larva. *Kaibogaku Zasshi J Anat* 69, 1–13.

Hotta, K., Mitsuhara, K., Takahashi, H., Inaba, K., Oka, K., Gojobori, T., et al. (2007). A web-based interactive developmental table for the ascidian Ciona intestinalis, including 3D real-image

embryo reconstructions: I. From fertilized egg to hatching larva. *Dev Dynam* 236, 1790–1805. doi:10.1002/dvdy.21188.

Hu, H., Uesaka, M., Guo, S., Shimai, K., Lu, T.-M., Li, F., et al. (2017). Constrained vertebrate evolution by pleiotropic genes. *Nature Ecology & Evolution* 1, 1722–1730. doi:10.1038/s41559-017-0318-0.

Huber, W., Carey, V. J., Gentleman, R., Anders, S., Carlson, M., Carvalho, B. S., et al. (2015). Orchestrating high-throughput genomic analysis with Bioconductor. *Nature Methods* 12, 115–121. doi:10.1038/nmeth.3252.

Hunter, J. D. (2007). Matplotlib: a 2D graphics environment. *Comput Sci Eng* 9, 90–95. doi:10.1109/mcse.2007.55.

Ichikawa, K., Tomioka, S., Suzuki, Y., Nakamura, R., Doi, K., Yoshimura, J., et al. (2017). Centromere evolution and CpG methylation during vertebrate speciation. *Nat Commun* 8, 1833. doi:10.1038/s41467-017-01982-7.

Kaufman, M. H. (1992). *The Atlas of Mouse Development*. Academic Press.

Kim, D., Paggi, J. M., Park, C., Bennett, C., and Salzberg, S. L. (2019). Graph-based genome alignment and genotyping with HISAT2 and HISAT-genotype. *Nat Biotechnol* 37, 907–915. doi:10.1038/s41587-019-0201-4.

Kimmel, C. B., Ballard, W. W., Kimmel, S. R., Ullmann, B., and Schilling, T. F. (1995). Stages of embryonic development of the zebrafish. *Dev Dynam* 203, 253–310. doi:10.1002/aja.1002030302.

Kinoshita, M., Murata, K., Naruse, K., and Tanaka, M. (2012). Medaka: Biology, Management, and Experimental Protocols. 165–275. doi:10.1002/9780813818849.ch6.

Klopfenstein, D. V., Zhang, L., Pedersen, B. S., Ramírez, F., Vesztrocy, A. W., Naldi, A., et al. (2018). GOATOOLS: a Python library for Gene Ontology analyses. *Scientific Reports* 8, 10872–17. doi:10.1038/s41598-018-28948-z.

Lefort, V., Desper, R., and Gascuel, O. (2015). FastME 2.0: a comprehensive, accurate, and fast distance-based phylogeny inference program. *Mol Biol Evol* 32, 2798–2800. doi:10.1093/molbev/msv150.

Li, H., Handsaker, B., Wysoker, A., Fennell, T., Ruan, J., Homer, N., et al. (2009). The Sequence Alignment/Map format and SAMtools. *Bioinformatics* 25, 2078–2079. doi:10.1093/bioinformatics/btp352.

Li, L., Jr, C. J. S., and Roos, D. S. (2003). OrthoMCL: Identification of ortholog groups for eukaryotic genomes. *Genome Research* 13, 2178–2189. doi:10.1101/gr.1224503.

Li, Y., Kikuchi, M., Li, X., Gao, Q., Xiong, Z., Ren, Y., et al. (2018). Weighted gene co-expression network analysis reveals potential genes involved in early metamorphosis process in sea

cucumber Apostichopus japonicus. *Biochemical and Biophysical Research Communications* 495, 1395–1402. doi:10.1016/j.bbrc.2017.11.154.

Li, Y., Omori, A., Flores, R. L., Satterfield, S., Nguyen, C., Ota, T., et al. (2020). Genomic insights of body plan transitions from bilateral to pentameral symmetry in Echinoderms. *Commun Biology* 3, 371. doi:10.1038/s42003-020-1091-1.

McKerns, M. M., Strand, L., Sullivan, T., Fang, A., and Aivazis, M. A. G. (2012). Building a framework for predictive science. *Arxiv*.

Mortazavi, A., Williams, B. A., McCue, K., Schaeffer, L., and Wold, B. (2008). Mapping and quantifying mammalian transcriptomes by RNA-Seq. *Nat Methods* 5, 621–628. doi:10.1038/nmeth.1226.

Neph, S., Kuehn, M. S., Reynolds, A. P., Haugen, E., Thurman, R. E., Johnson, A. K., et al. (2012). BEDOPS: high-performance genomic feature operations. *Bioinformatics* 28, 1919–1920. doi:10.1093/bioinformatics/bts277.

Nieuwkoop, P. D., and Faber, J. (1994). *Normal Table of Xenopus laevis (Daudin)*. New York: Garland Science.

Paradis, E., and Schliep, K. (2018). ape 5.0: an environment for modern phylogenetics and evolutionary analyses in R. *Bioinformatics* 35, 526–528. doi:10.1093/bioinformatics/bty633.

Patil, I. (2021). Visualizations with statistical details: the “ggstatsplot” approach. *J Open Source Softw* 6, 3167. doi:10.21105/joss.03167.

Pertea, M., Pertea, G. M., Antonescu, C. M., Chang, T.-C., Mendell, J. T., and Salzberg, S. L. (2015). StringTie enables improved reconstruction of a transcriptome from RNA-seq reads. *Nat Biotechnol* 33, 290–295. doi:10.1038/nbt.3122.

Plotly (2015). Collaborative data science. Available at: https://plot.ly.

Prince, V. E., Joly, L., Ekker, M., and Ho, R. K. (1998). Zebrafish hox genes: genomic organization and modified colinear expression patterns in the trunk. *Dev Camb Engl* 125, 407–20.

Quinlan, A. R., and Hall, I. M. (2010). BEDTools: a flexible suite of utilities for comparing genomic features. *Bioinformatics* 26, 841–842. doi:10.1093/bioinformatics/btq033.

Revell, L. J. (2011). phytools: an R package for phylogenetic comparative biology (and other things). *Methods in Ecology and Evolution* 3, 217–223. doi:10.1111/j.2041-210x.2011.00169.x.

Revell, L. J., and Chamberlain, S. A. (2014). Rphylip: an R interface for PHYLIP. *Methods Ecol Evol* 5, 976–981. doi:10.1111/2041-210x.12233.

Saitou, N., and Nei, M. (1987). The neighbor-joining method: a new method for reconstructing phylogenetic trees. *Mol Biol Evol*. doi:10.1093/oxfordjournals.molbev.a040454.

Tabari, E., and Su, Z. (2017). PorthoMCL: Parallel orthology prediction using MCL for the realm of massive genome availability. *Big Data Analytics* 2, 4. doi:10.1186/s41044-016-0019-8.

Talevich, E., Invergo, B. M., Cock, P. J., and Chapman, B. A. (2012). Bio.Phylo: a unified toolkit for processing, analyzing and visualizing phylogenetic trees in Biopython. *Bmc Bioinformatics* 13, 209. doi:10.1186/1471-2105-13-209.

Tokita, M., and Kuratani, S. (2001). Normal embryonic stages of the Chinese softshelled turtle Pelodiscus sinensis (Trionychidae). *Zool Sci* 18, 705–715. doi:10.2108/zsj.18.705.

Törönen, P., Medlar, A., and Holm, L. (2018). PANNZER2: a rapid functional annotation web server. *Nucleic Acids Res* 46, gky350-. doi:10.1093/nar/gky350.

Trapnell, C., Williams, B. A., Pertea, G., Mortazavi, A., Kwan, G., Baren, M. J. van, et al. (2010). Transcript assembly and quantification by RNA-Seq reveals unannotated transcripts and isoform switching during cell differentiation. *Nat Biotechnol* 28, 511–515. doi:10.1038/nbt.1621.

Tu, Q., Cameron, R. A., and Davidson, E. H. (2014). Quantitative developmental transcriptomes of the sea urchin Strongylocentrotus purpuratus. *Dev Biol* 385, 160–167. doi:10.1016/j.ydbio.2013.11.019.

Tu, Q., Cameron, R. A., Worley, K. C., Gibbs, R. A., and Davidson, E. H. (2012). Gene structure in the sea urchin Strongylocentrotus purpuratus based on transcriptome analysis. *Genome Res* 22, 2079–2087. doi:10.1101/gr.139170.112.

Virtanen, P., Gommers, R., Oliphant, T. E., Haberland, M., Reddy, T., Cournapeau, D., et al. (2020). SciPy 1.0: fundamental algorithms for scientific computing in Python. *Nat Methods* 17, 261–272. doi:10.1038/s41592-019-0686-2.

Walt, S. van der, Schönberger, J. L., Nunez-Iglesias, J., Boulogne, F., Warner, J. D., Yager, N., et al. (2014). scikit-image: image processing in Python. *Peerj* 2, e453. doi:10.7717/peerj.453.

Wang, Z., Pascual-Anaya, J., Zadissa, A., Li, W., Niimura, Y., Huang, Z., et al. (2013). The draft genomes of soft-shell turtle and green sea turtle yield insights into the development and evolution of the turtle-specific body plan. *Nat Genet* 45, 701–706. doi:10.1038/ng.2615.

Waskom, M. (2021). seaborn: statistical data visualization. *J Open Source Softw* 6, 3021. doi:10.21105/joss.03021.

Wickham, H. (2016). *ggplot2: Elegant Graphics for Data Analysis*. Springer-Verlag New York Available at: https://ggplot2.tidyverse.org.

Yu, G., Smith, D. K., Zhu, H., Guan, Y., and Lam, T. T.-Y. (2016). ggtree: an R package for visualization and annotation of phylogenetic trees with their covariates and other associated data. *Methods in Ecology and Evolution* 8, 28–36. doi:10.1111/2041-210x.12628.

Yu, J. K. S., and Holland, L. Z. (2009). Cephalochordates (amphioxus or lancelets): a model for understanding the evolution of chordate characters. *Cold Spring Harb Protoc* 2009, pdb.emo130-pdb.emo130. doi:10.1101/pdb.emo130.

Zhang, G., Fang, X., Guo, X., Li, L., Luo, R., Xu, F., et al. (2012). The oyster genome reveals stress adaptation and complexity of shell formation. *Nature* 490, 49–54. doi:10.1038/nature11413.
